# Supplementary material for: Discovery and rational engineering of PET hydrolase with both mesophilic and thermophilic PET hydrolase properties
Source: Nat Commun. 2023 Jul 28;14:4556. doi: 10.1038/s41467-023-40233-w (PMC10382486; doi:10.1038/s41467-023-40233-w)
Supplement: Supplementary file 1 — Supplementary information [file 41467_2023_40233_MOESM1_ESM.pdf]

Supplementary Information

Discovery and rational engineering of PET hydrolase with both mesophilic and thermophilic PET hydrolase properties

Hwaseok Hong[1,4], Dongwoo Ki[1,4], Hogyun Seo[1], Jiyoung Park[1], Jaewon Jang[2], and Kyung-Jin Kim[1,3,*]

[1]School of Life Sciences, BK21 FOUR KNU Creative BioResearch Group, KNU Institute for Microorganisms, Kyungpook National University, Daegu 41566, Republic of Korea

[2]Bioresearch Research Institute, CJ CheilJedang Co., Suwon 16495, Republic of Korea

[3]Zyen Co, Daegu 41566, Republic of Korea

[4]These authors contributed equally to this work.

***Correspondence**: kkim@knu.ac.kr (K-J. Kim)

Kyung-Jin Kim, Ph.D.

School of Life Sciences, BK21 FOUR KNU Creative BioResearch Group, KNU Institute for Microorganisms, Kyungpook National University, Daegu 41566, Republic of Korea

Tel: +82-53-950-5377, Fax: +82-53-955-5522, Email: kkim@knu.ac.kr

This PDF file includes:

Supplementary Figs 1 to 31.

Supplementary Tables 1 to 4.

Table of Contents

Supplementary Figures ....

Supplementary Fig. 1 3
Supplementary Fig. 2 6
Supplementary Fig. 3 7
Supplementary Fig. 4 8
Supplementary Fig. 5 9
Supplementary Fig. 6 10
Supplementary Fig. 7 11
Supplementary Fig. 8 12
Supplementary Fig. 9 13
Supplementary Fig. 10 14
Supplementary Fig. 11 15
Supplementary Fig. 12 16
Supplementary Fig. 13 17
Supplementary Fig. 14 18
Supplementary Fig. 15 19
Supplementary Fig. 16 20
Supplementary Fig. 17 21
Supplementary Fig. 18 22
Supplementary Fig. 19 23
Supplementary Fig. 20 24
Supplementary Fig. 21 25
Supplementary Fig. 22 26
Supplementary Fig. 23 27
Supplementary Fig. 24 28
Supplementary Fig. 25 29
Supplementary Fig. 26 30
Supplementary Fig. 27 31
Supplementary Fig. 28 32
Supplementary Fig. 29 33
Supplementary Fig. 30 34
Supplementary Fig. 31 38

Supplementary Tables ....

Supplementary Table S1 39
Supplementary Table S2 40
Supplementary Table S3 41
Supplementary Table S4 42

Supplementary References ....

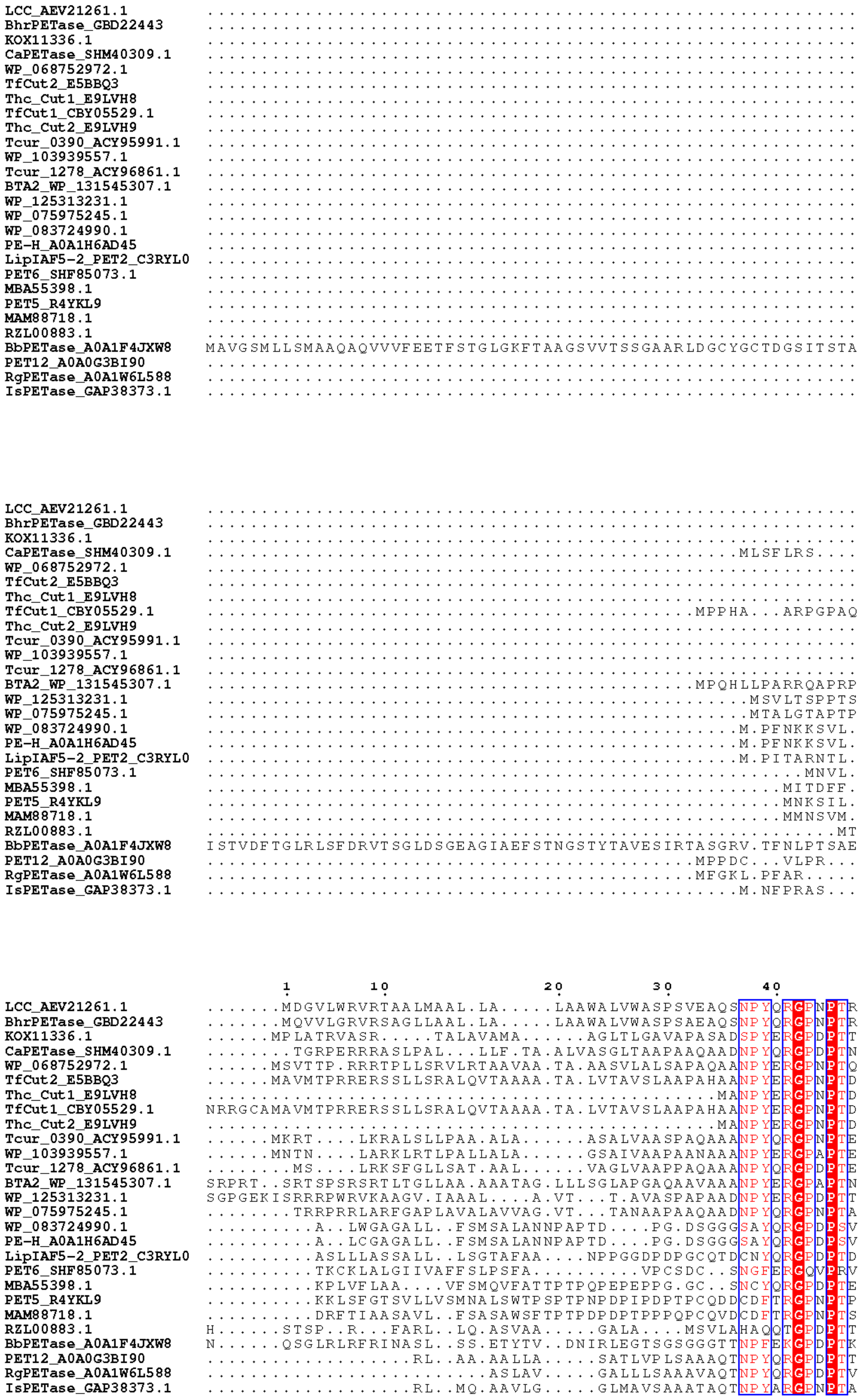

Supplementary Fig. 1. Sequence alignment of enzymes used in phylogenetic tree analysis.

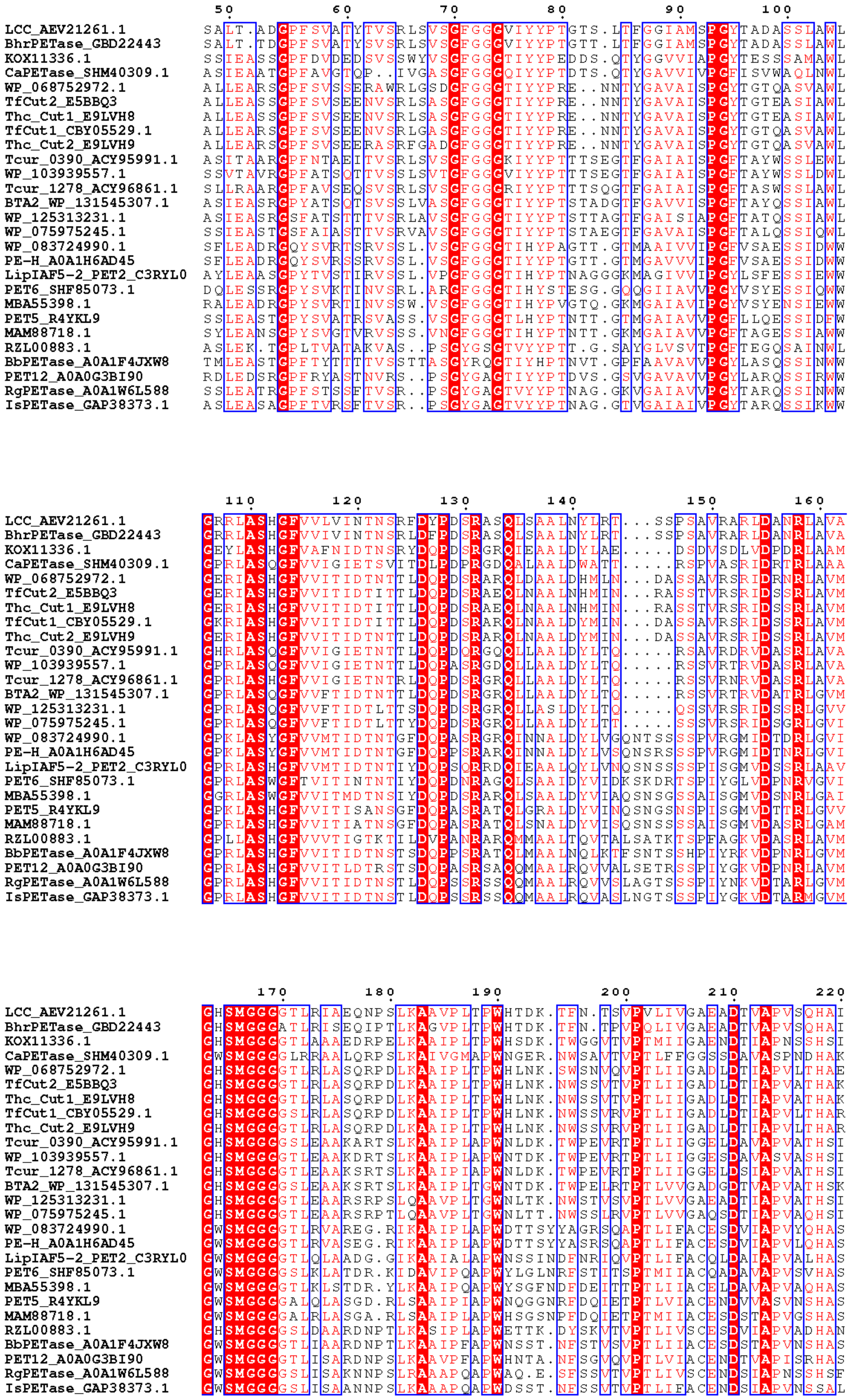

Supplementary Fig. 1. Sequence alignment of enzymes used in phylogenetic tree analysis. (continued)

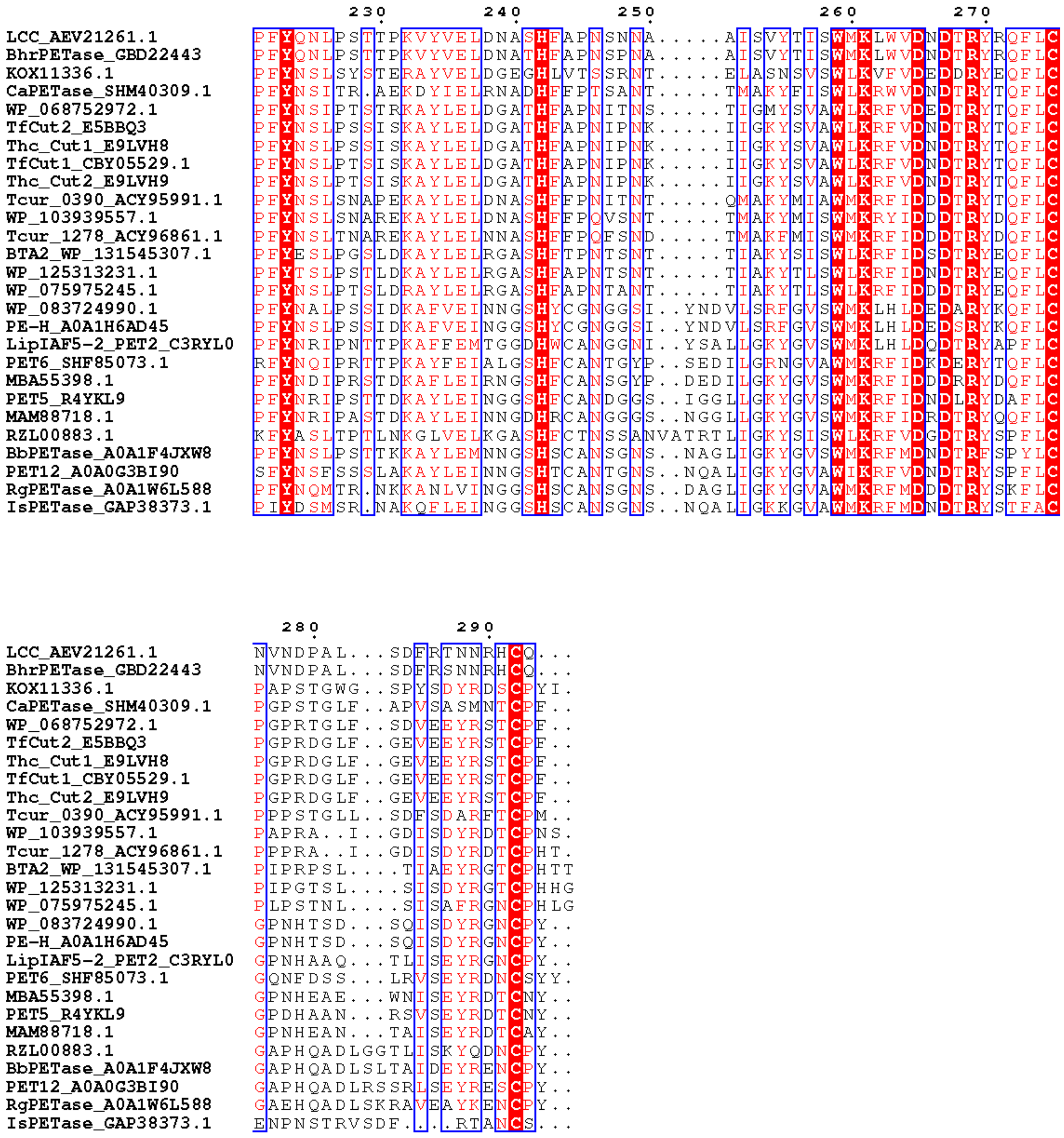

Supplementary Fig. 1. Sequence alignment of enzymes used in phylogenetic tree analysis. (continued)

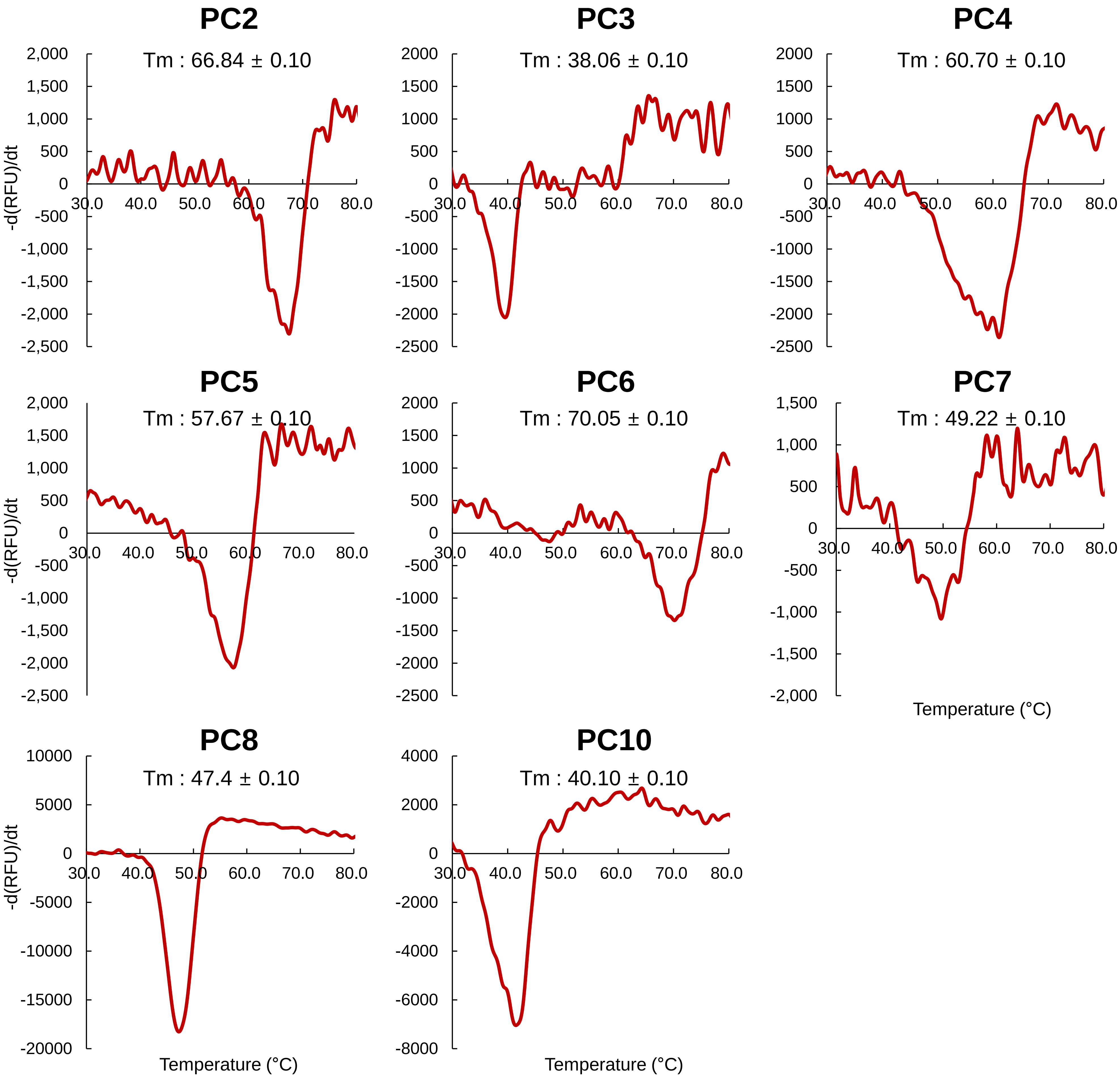

Supplementary Fig. 2. Thermostability of 8 selected PETase candidates (PCs) determined by differential scanning fluorimetry (DSF). Melting curves and melting points of PC2-PC8, and PC10. Tm values are presented as mean values ± SD, n=2.

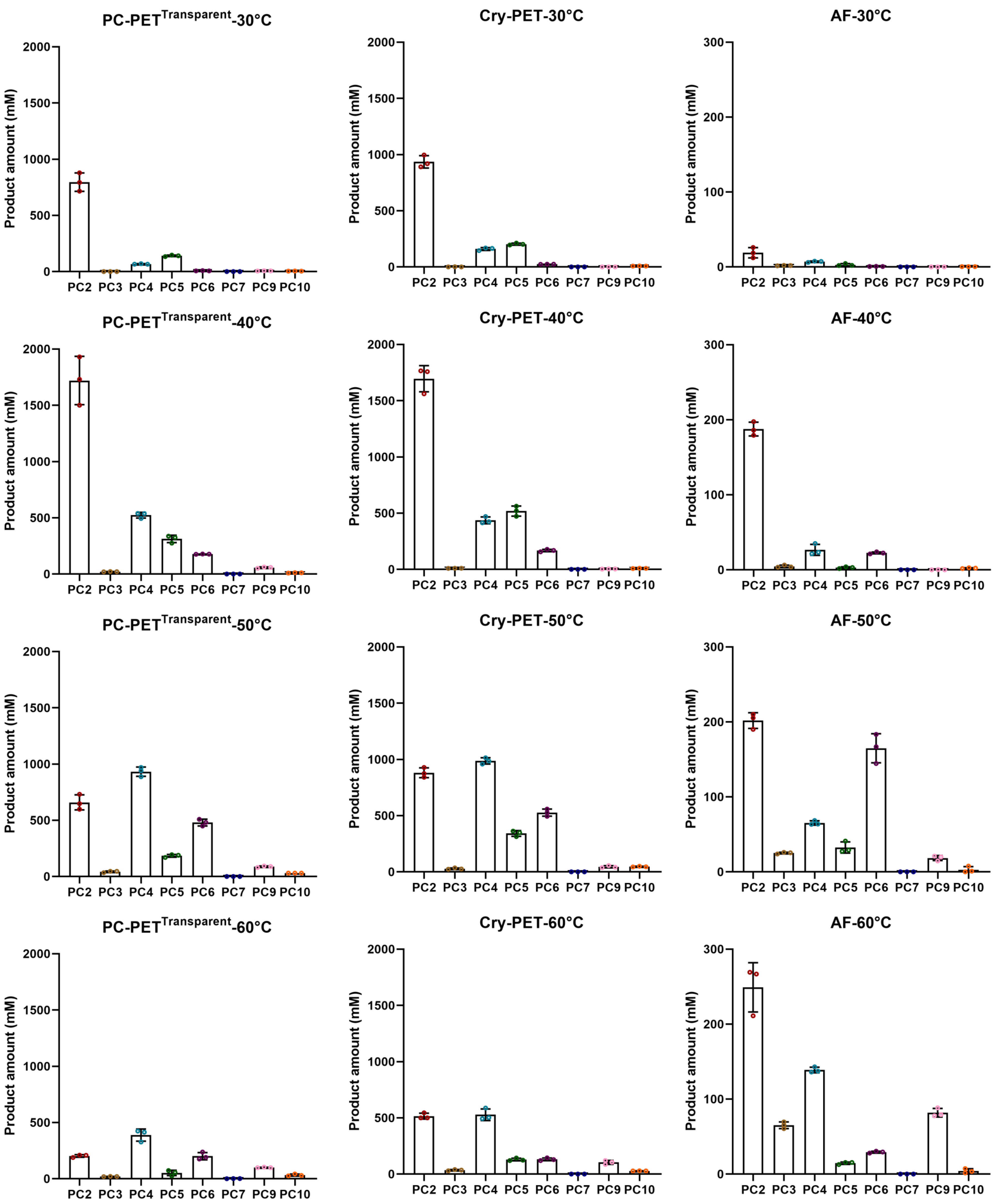

Supplementary Fig. 3. PET hydrolytic activity of the eight PCs. The reaction was performed with PC-PET^{Transparent} (15 mg mL^{-1} with 500 nM enzyme), Cry-PET (15 mg mL^{-1} with 2 μM enzyme), and AF-PET (15 mg mL^{-1} with 2 μM enzyme) in 50 mM Glycine-NaOH pH 9.0 buffer at various temperatures (30 °C, 40 °C, 50 °C, 60 °C) for 3 days. Reactions were performed in triplicate; Data are presented as mean values ± SD.

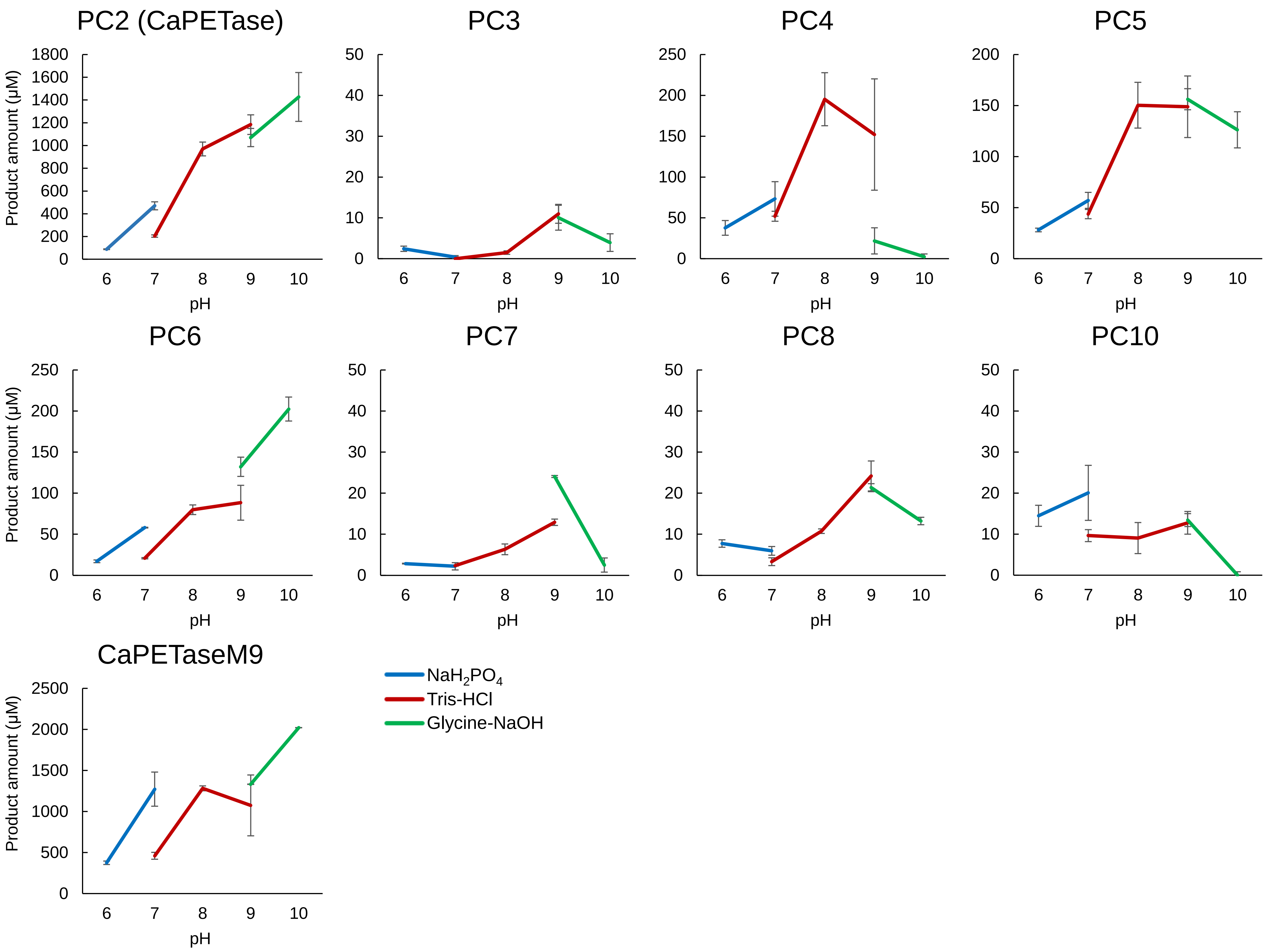

Supplementary Fig. 4. The pH profiles of PC2-PC8, PC10, and CaPETase^{M9} enzymes involved in this study. The PET hydrolytic products released by PC2-PC8, PC10, and CaPETase^{M9} enzymes were measured in 50 mM sodium phosphate buffer (pH 6-7), Tris-HCl buffer (pH 8-9), and 50 mM glycine-NaOH buffer (pH 9-10) at 30ºC after 3 days, using PC-PET as the substrate. Reactions were performed in triplicate; Data are presented as mean values ± SD.

a

b

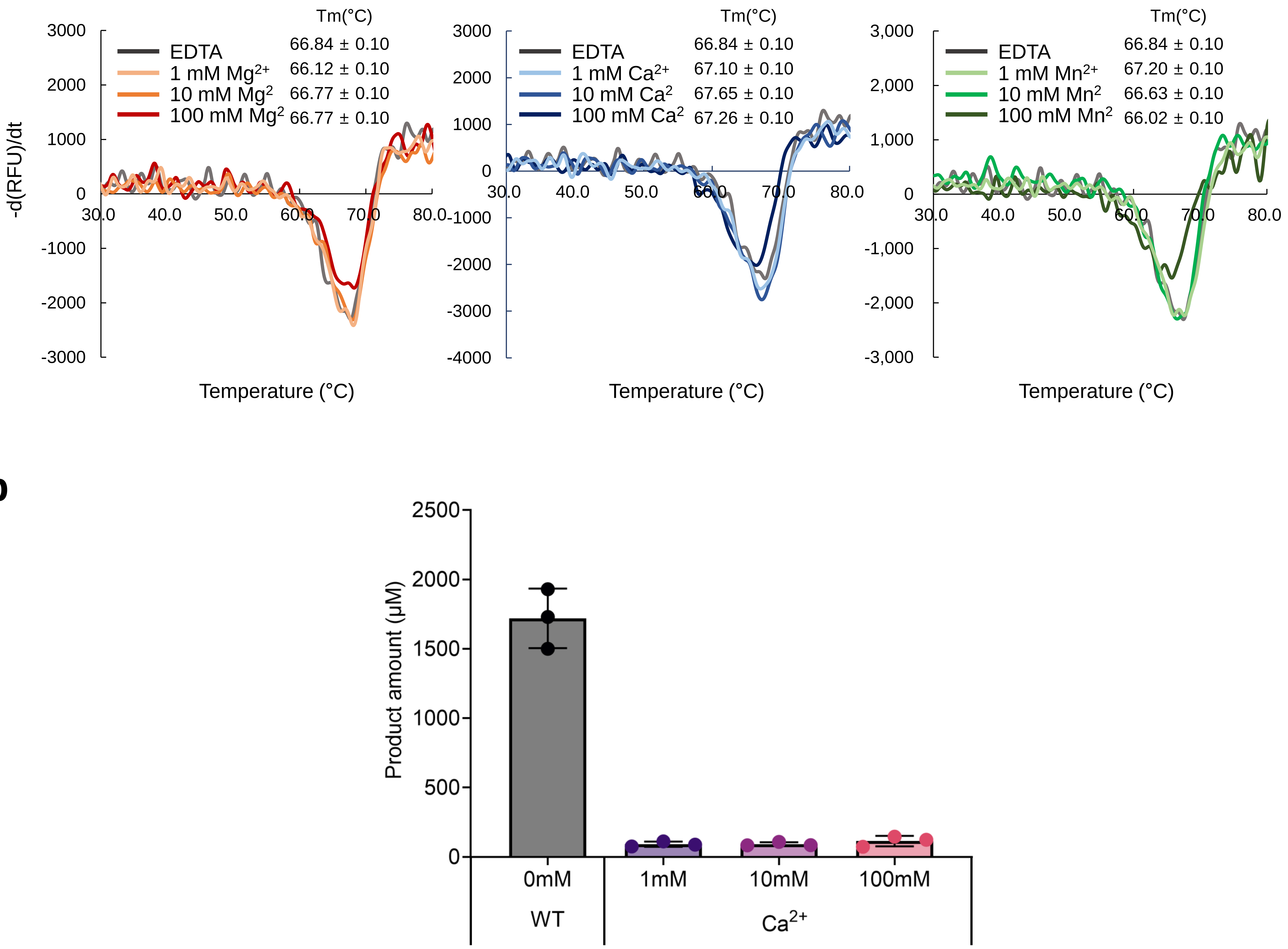

Supplementary Fig. 5. Effect of metal ions on thermostability and PET hydrolytic activity of PC2. (a) Thermostability of PC2 determined by differential scanning fluorimetry in the presence of $MgCl_2$, $CaCl_2$, and $MnSO_4$ (0 mM, 1 mM or 100 mM). (b) The PET hydrolytic activity of PC2 in in the presence of $CaCl_2$ (0 mM, 1 mM or 100 mM). The reaction was performed in 50 mM Glycine-NaOH buffer pH 9.0 at 40ºC after 3 days, using PC-PET as the substrate. Reactions were performed in triplicate; Data are presented as mean values ± SD.

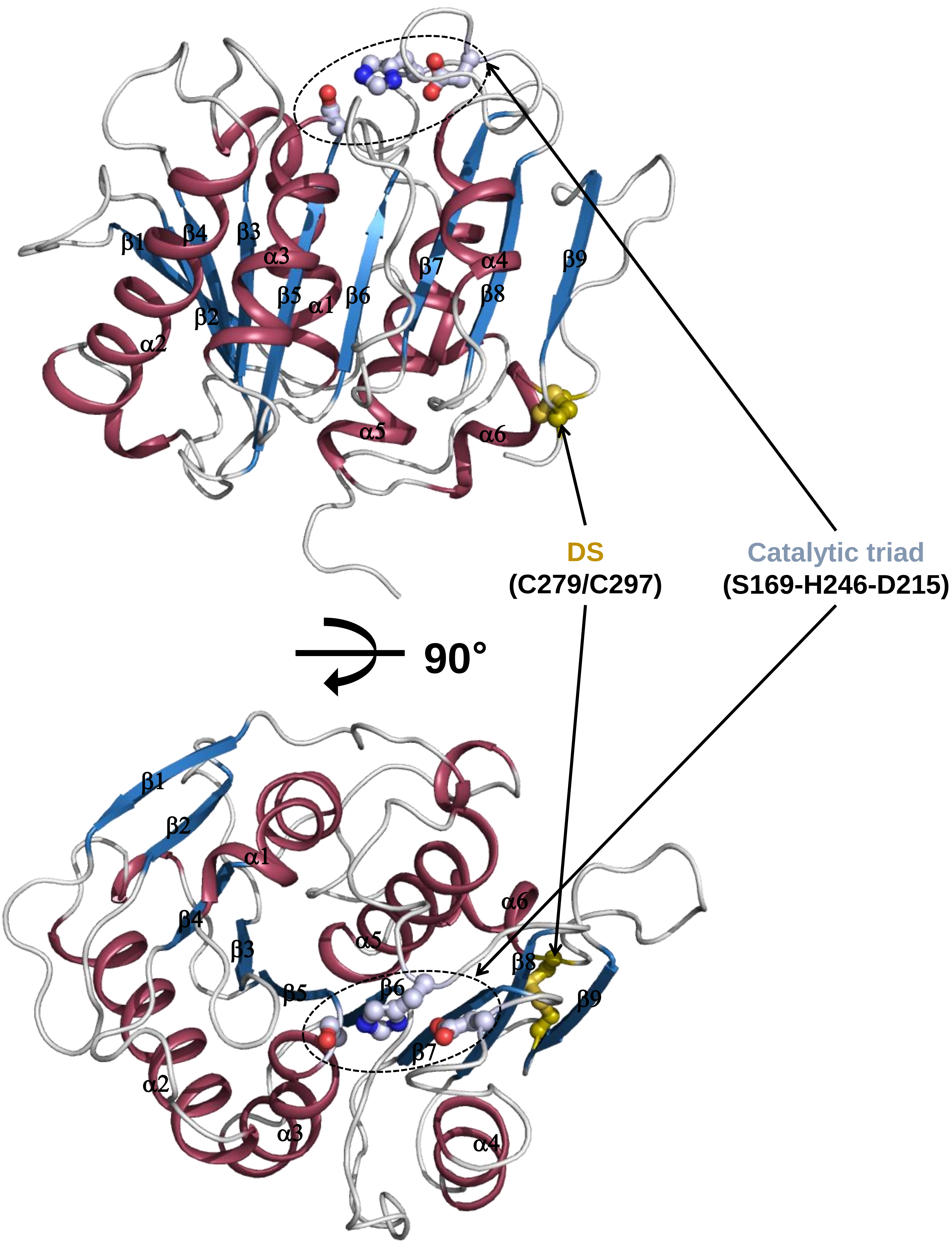

Supplementary Fig. 6. Crystal structure of *Ca*PETase.

	Catalytic triad			Wobbling tryptophan pairs			Residues forming the substrate binding cleft																	
Position in *Ca*PETase	169	246	215	194	223	227	101	102	103	107	133	168	170	195	196	217	247	250	No. of DS	Extended loop
***Ca*PETase**	S	H	D	W	H	F	F	I	S	Q	L	W	M	N	G	V	F	T	1	
***Tf*Cut2**	S	H	D	W	H	F	Y	T	A	S	Q	H	M	H	L	I	F	N	1	
**LCC**	S	H	D	W	H	F	Y	T	A	S	Y	H	M	H	T	V	F	N	1	
***Is*PETase**	S	H	D	W	S	I	Y	T	G	S	Q	W	M	D	S	I	S	N	2	S242-Q247

Supplementary Fig. 7. Comparisons of active site-lining residues and key structural features of known PET hydrolases.

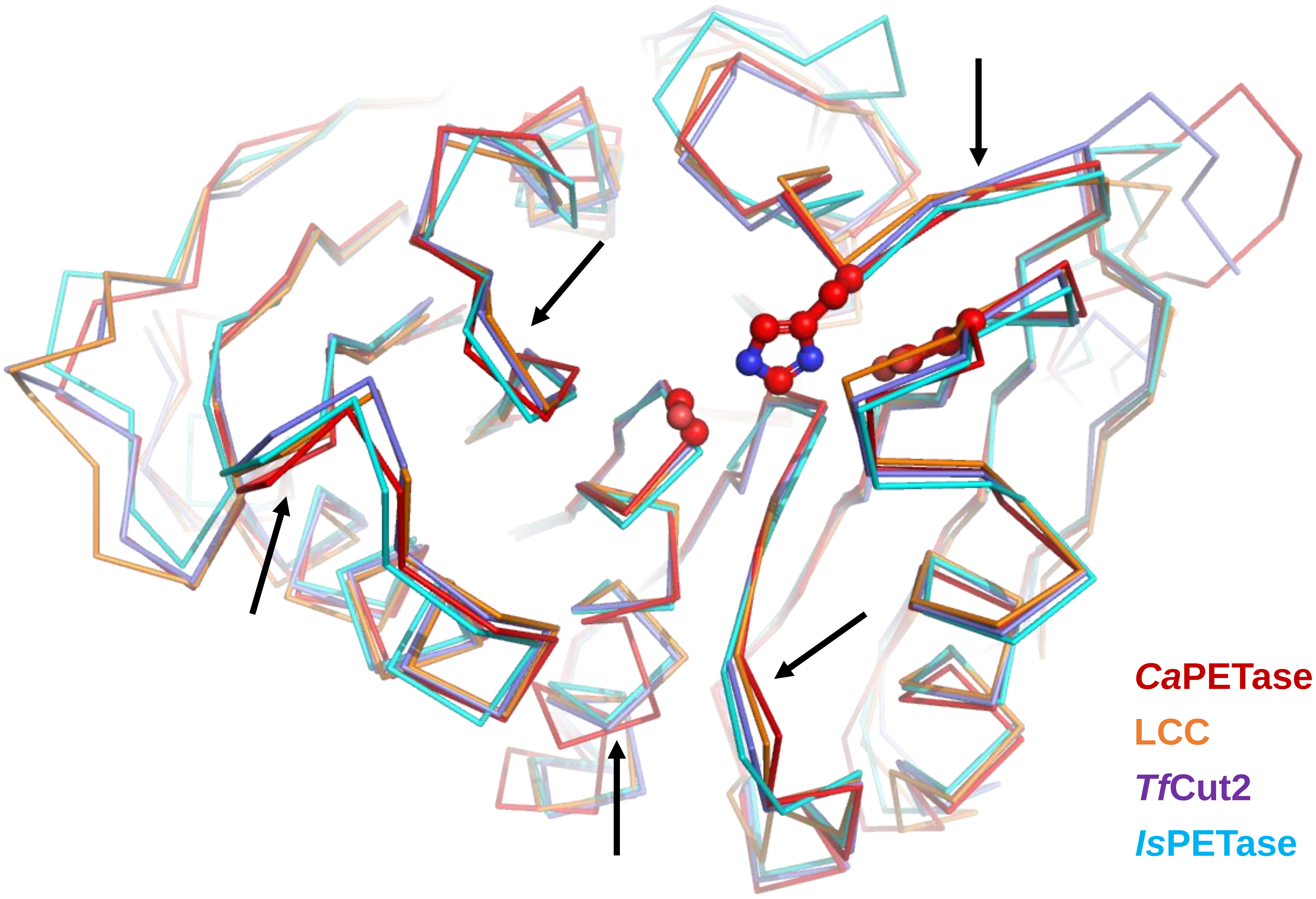

Supplementary Fig. 8. Superposition of the backbone representation of *Ca*PETase, LCC, *Tf*Cut2, and *Is*PETase. Structures of *Ca*PETase, LCC, *Tf*Cut2, and *Is*PETase are shown as ribbon models. Catalytic triad residues of *Ca*PETase are presented as a ball and stick model. The arrows denote regions with different backbone conformations between *Ca*PETase and the other three enzymes.

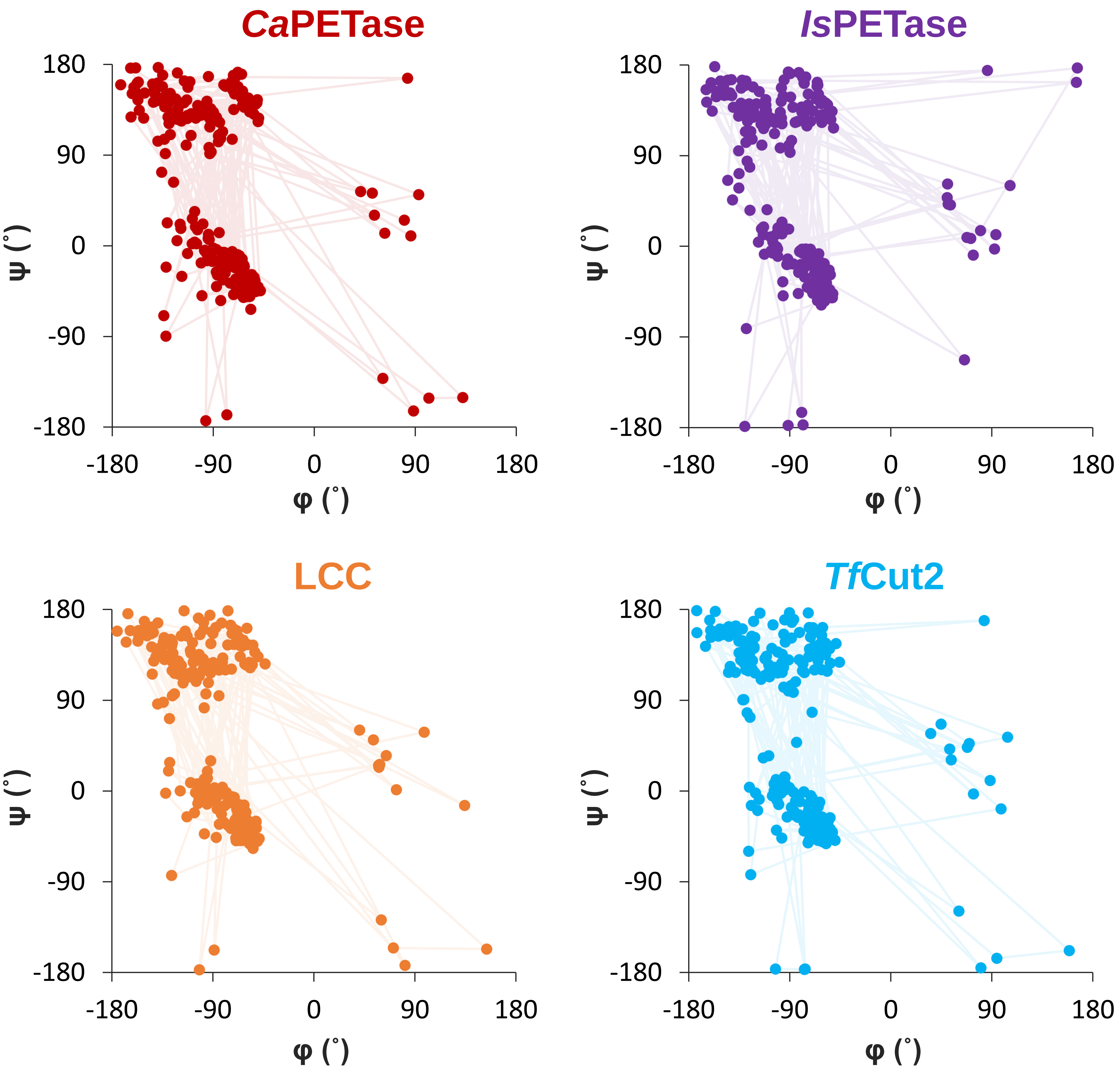

Supplementary Fig. 9. Ramachandran plots of *Ca*PETase, *Is*PETase, LCC, and *Tf*Cut2.

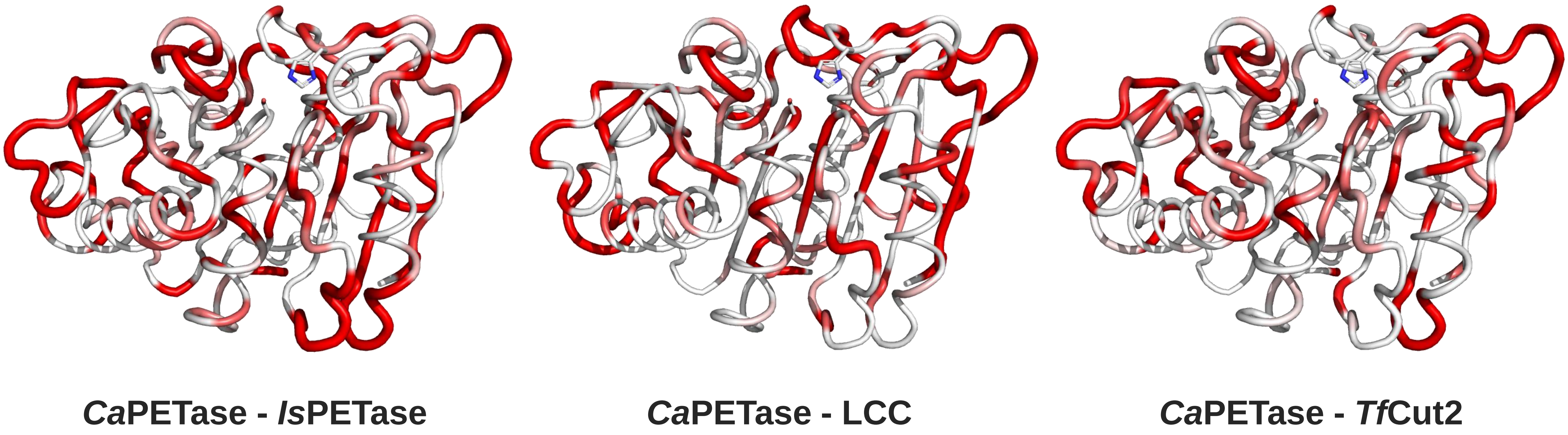

Supplementary Fig. 10. Differences of backbone torsion angle between *Ca*PETase and the other PET hydrolases. The overall structure of *Ca*PETase is displayed as a putty tube representation of the same diameter in PyMOL and is colored according to the Euclidean distance values between the two Ramachandran points of the aligned residues between *Ca*PETase and *Is*PETase, LCC, and *Tf*Cut2. Colors of white to red designate low to high Euclidean distance values, respectively. The catalytic triad of *Ca*PETase are shown as a stick model.

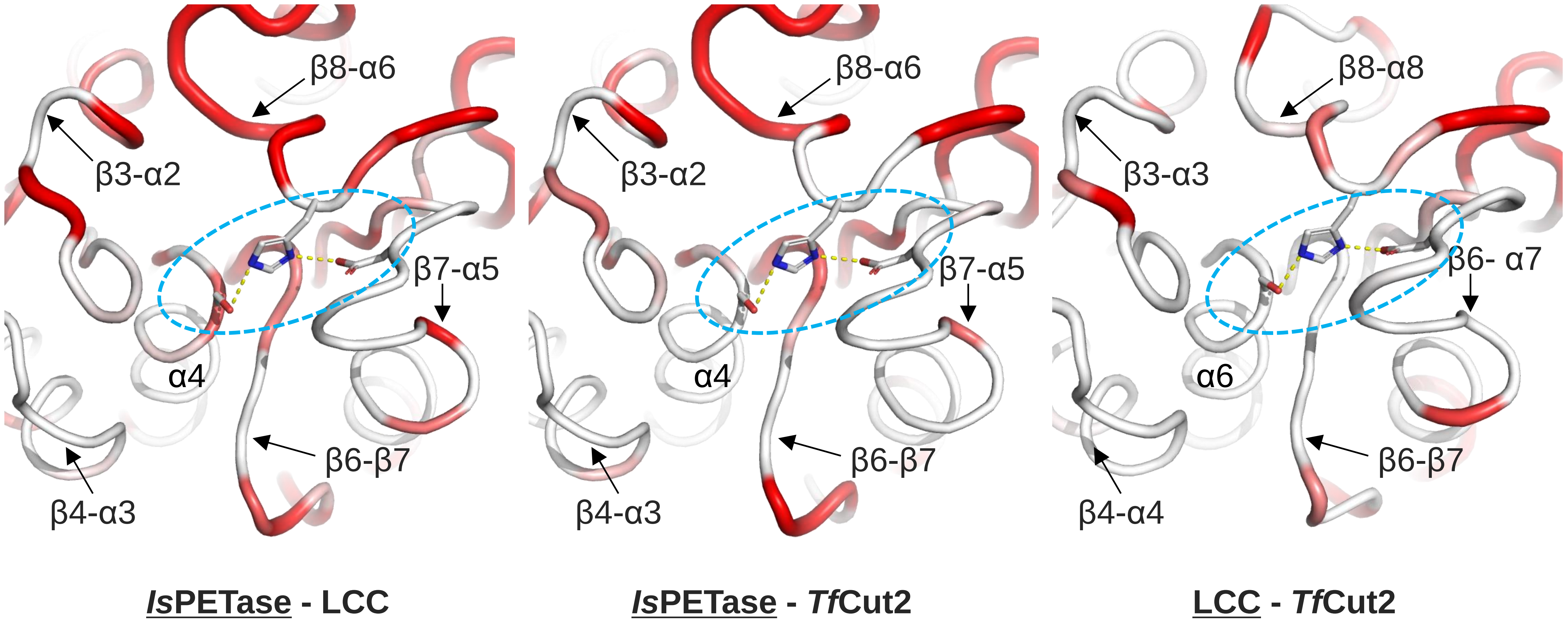

Supplementary Fig. 11. Comparisons of backbone torsion angle differences at the active site between *Is*PETase, LCC, and *Tf*Cut2. The structure of the underlined enzymes is displayed as a putty tube representation of the same diameter in PyMOL. The structure is colored according to the Euclidean distance values between the two Ramachandran points of the aligned residues. Colors of white to red designate low to high Euclidean distance values, respectively. The catalytic triad of each enzymes are shown as a stick model with a cyan-color circle.

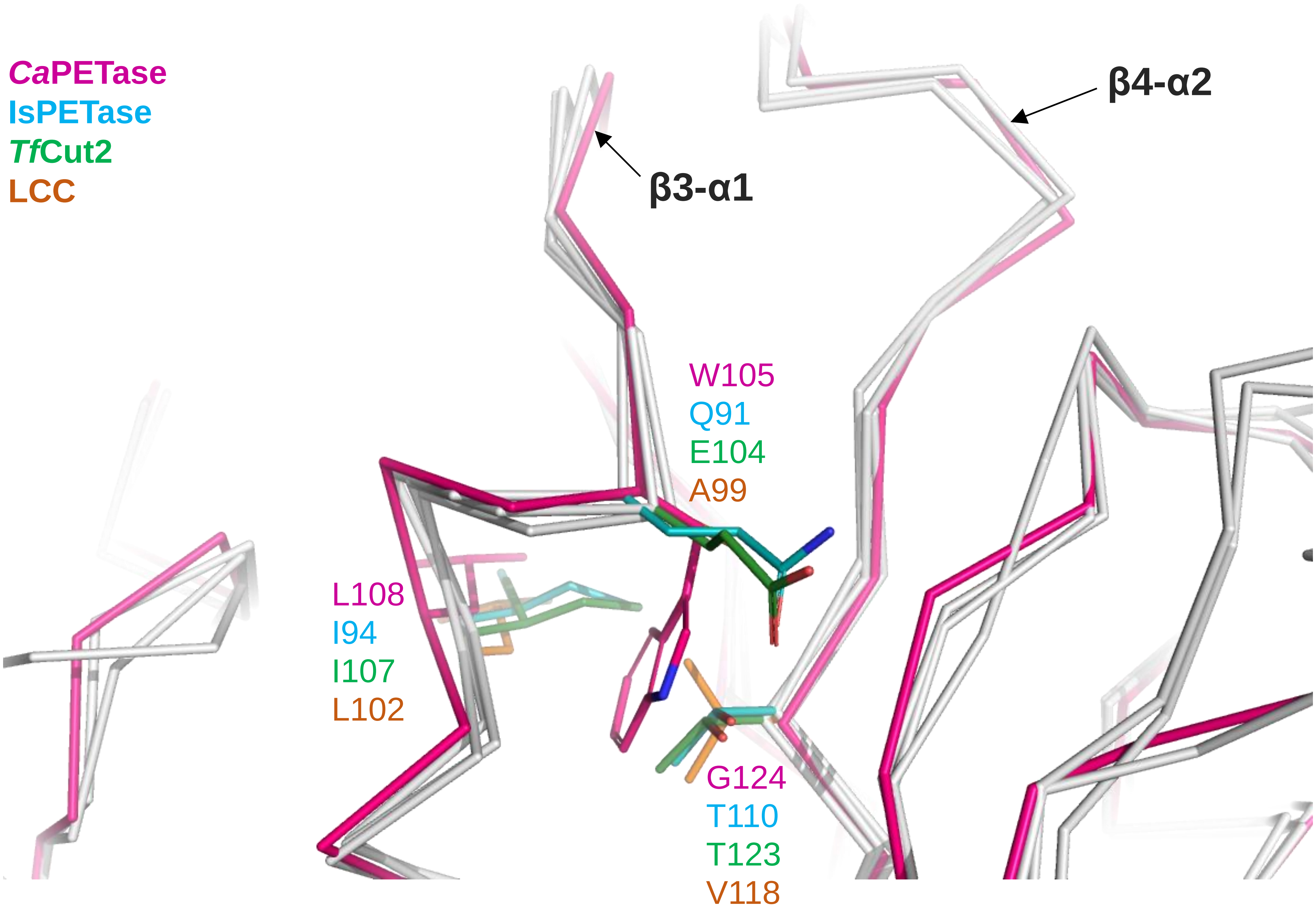

Supplementary Fig. 12. W105-L108-G124 local network of *Ca*PETase. Structures of *Ca*PETase, *Is*PETase, *Tf*Cut2, and LCC are shown as ribbon models. The residues forming the network of *Ca*PETase are shown as a stick model, and the corresponding residues of *Is*PETase, *Tf*Cut2, and LCC enzymes are also presented as a stick model.

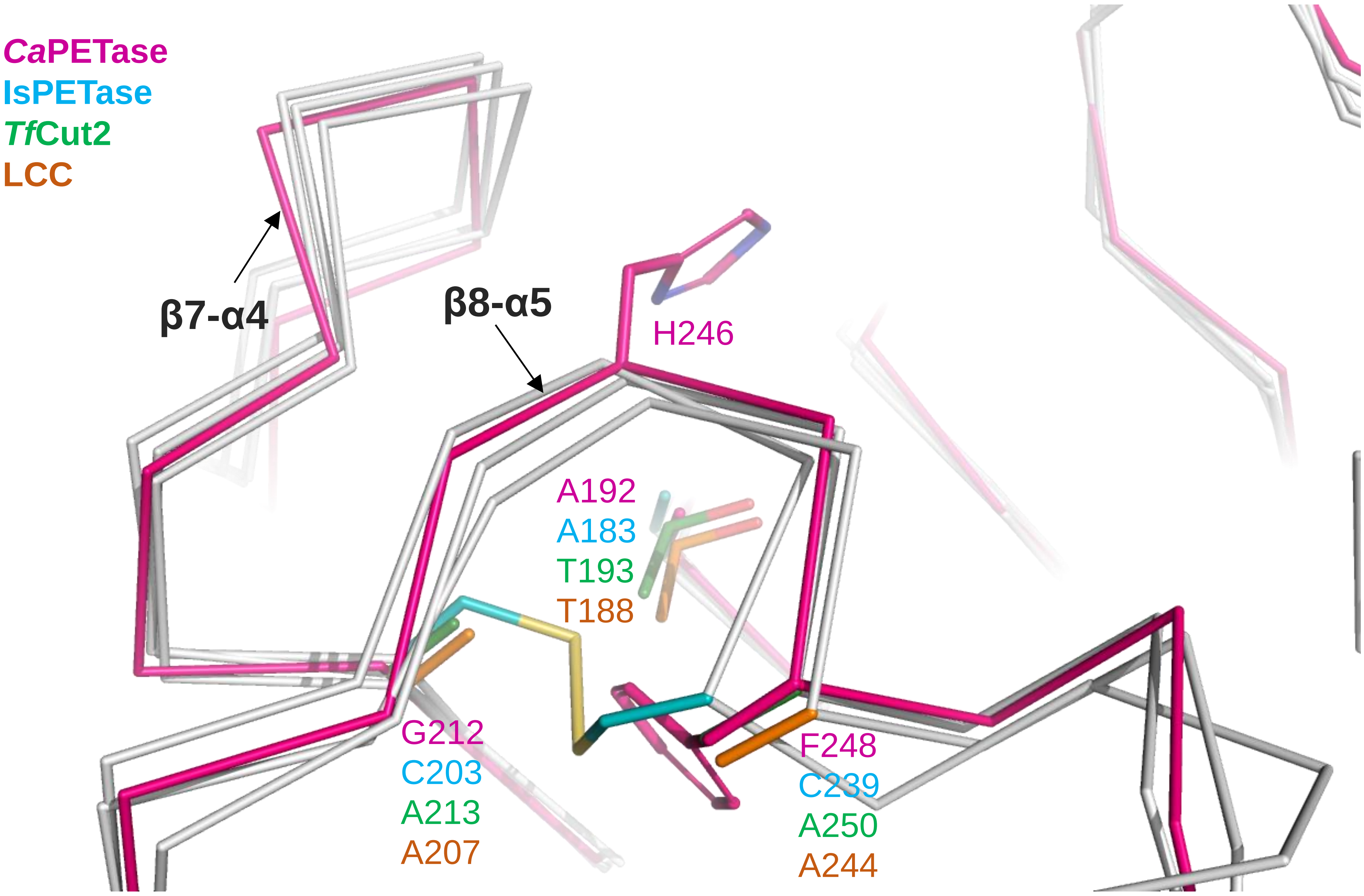

Supplementary Fig. 13. A192-G212-F248 local network of *Ca*PETase. Structures of *Ca*PETase, *Is*PETase, *Tf*Cut2, and LCC are shown as ribbon models. The residues forming the network and the catalytic histidine of *Ca*PETase are shown as a stick model, and the corresponding residues of *Is*PETase, *Tf*Cut2, and LCC enzymes are also presented as a stick model.

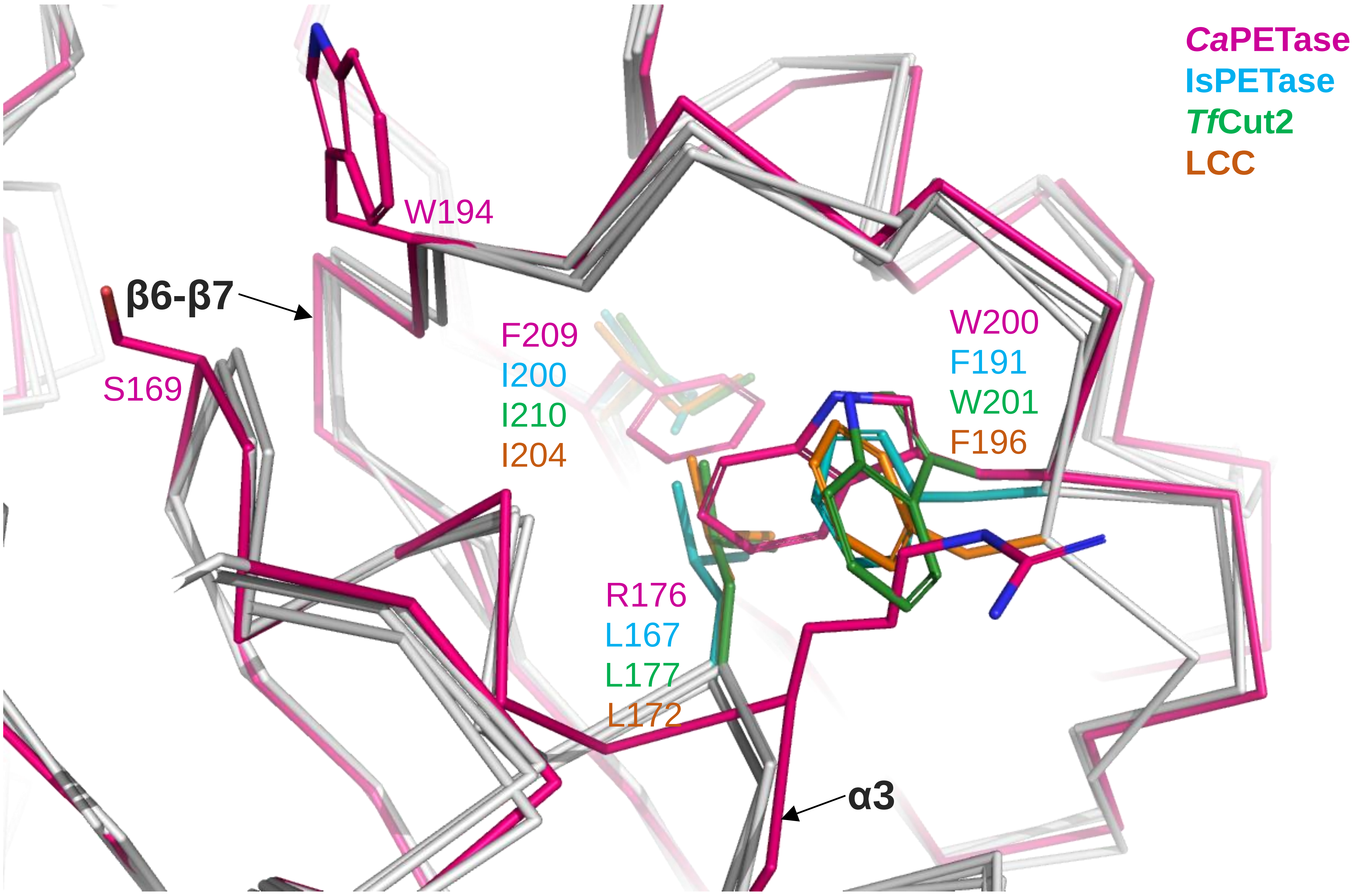

Supplementary Fig. 14. R176-W200-F209 local network of *Ca*PETase. Structures of *Ca*PETase, *Is*PETase, *Tf*Cut2, and LCC are shown as ribbon models. The residues forming the network and the wobbly tryptophan of *Ca*PETase are shown as a stick model, and the corresponding residues of *Is*PETase, *Tf*Cut2, and LCC enzymes are also presented as a stick model.

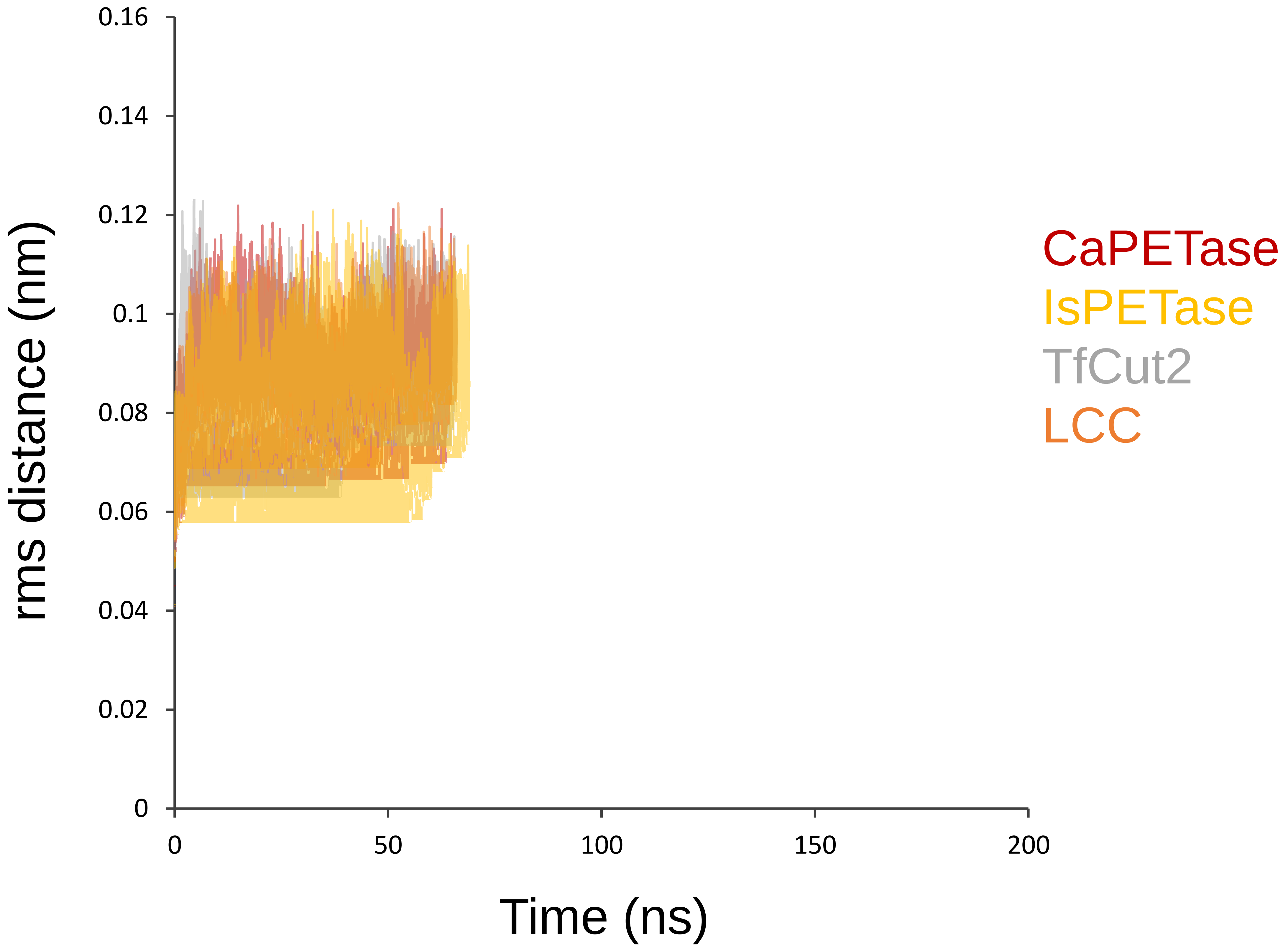

Supplementary Fig. 15. RMS distances during simulation between a trajectory and its crystal structure.

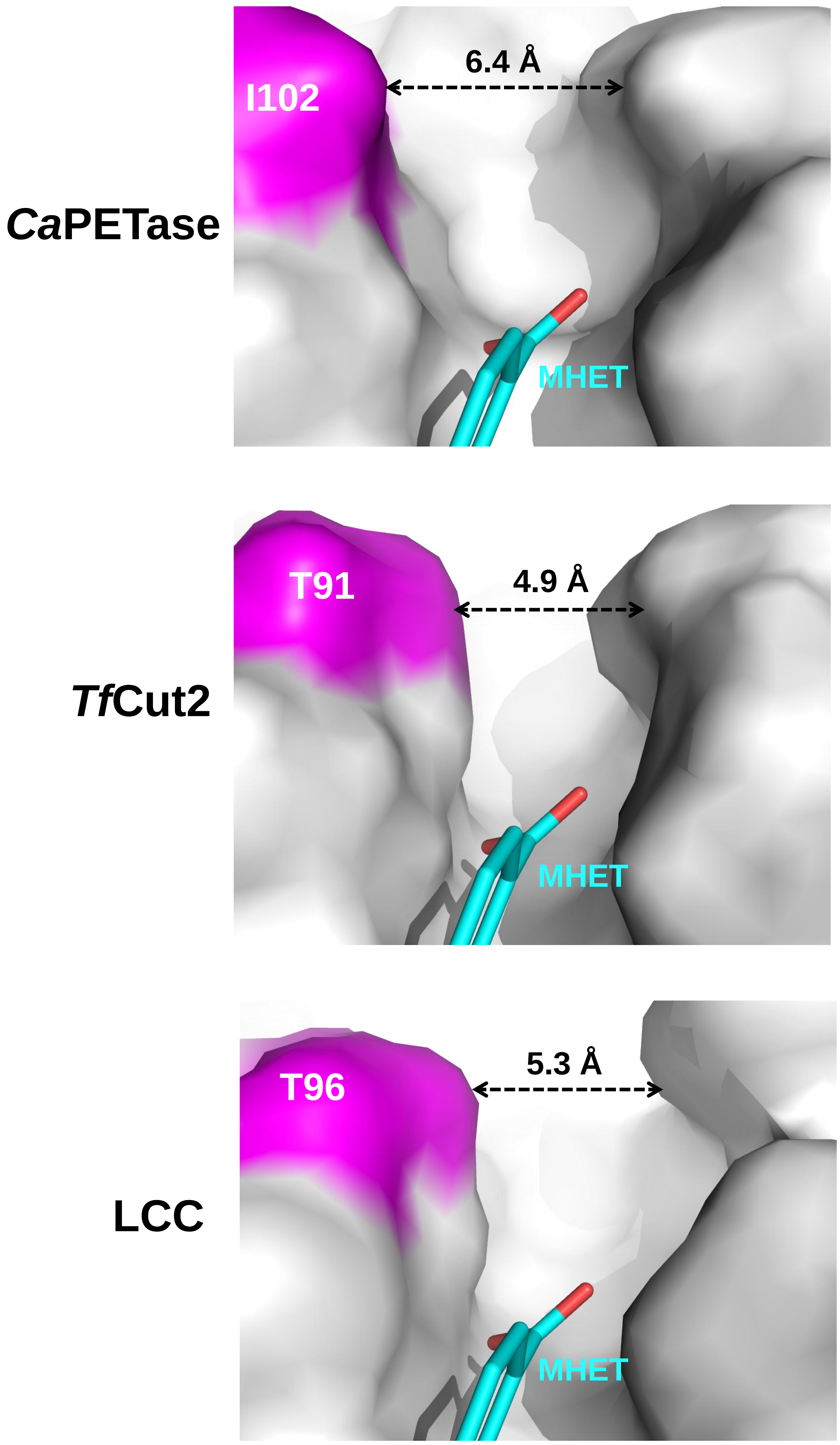

Supplementary Fig. 16. Comparison of the active site cleft neighboring I102 in *Ca*PETase, LCC, and *Tf*CUT2. The structures of *Ca*PETase, *Tf*Cut2, and LCC are presented as surface model with white color. I102 residues of *Ca*PETase and corresponding residues of *Tf*Cut2 and LCC are presented as a magenta color. Mono(2-hydroxyethly) terephthalate (MHET) molecule from LCC (PDB code: 7VVE) is presented as a stick model with cyan color.

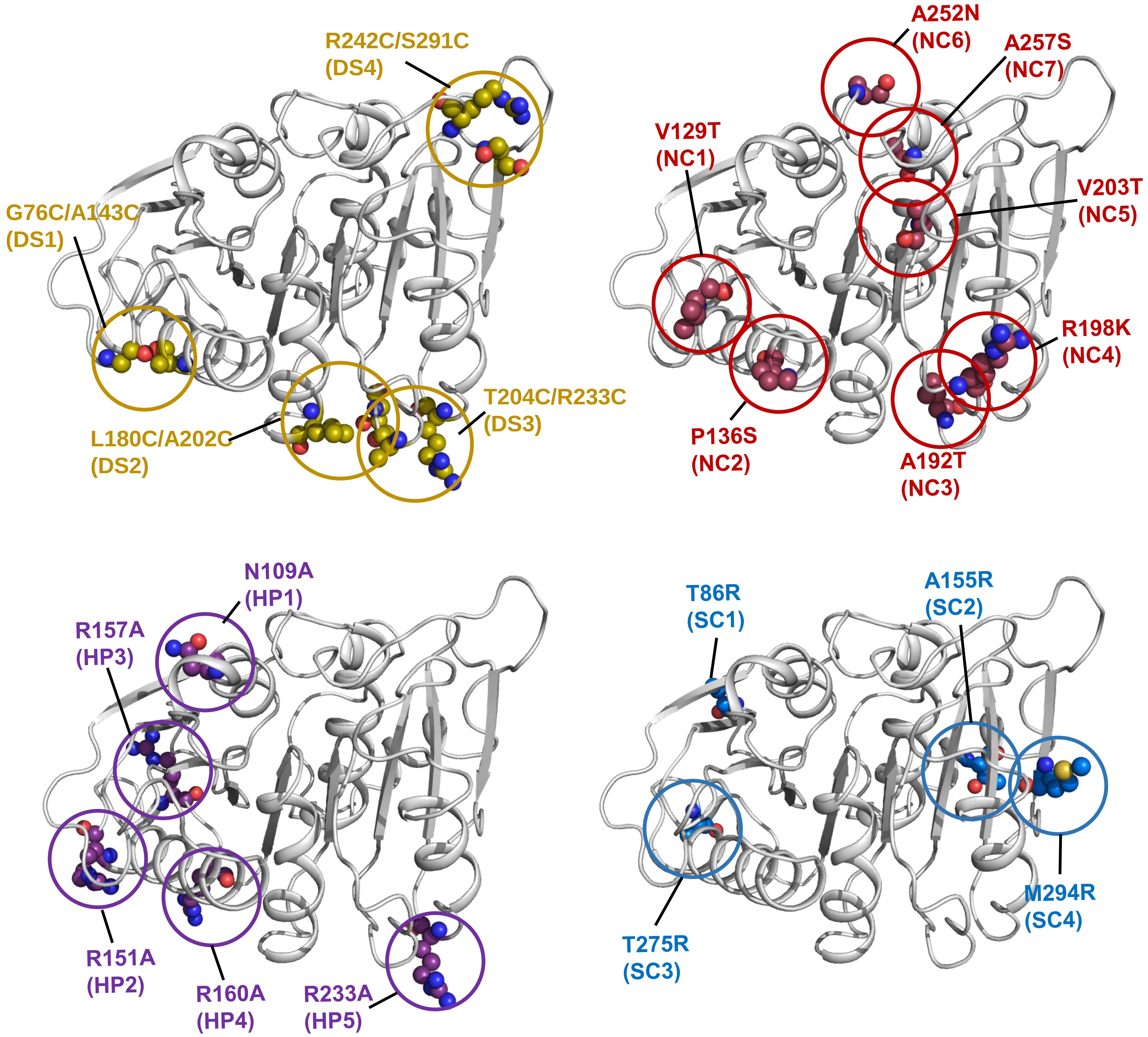

Supplementary Fig. 17. Mutation points of *Ca*PETase for rational engineering. Structures of *Ca*PETase are shown as grey-colored cartoon models and mutation points are presented as a ball and stick model.

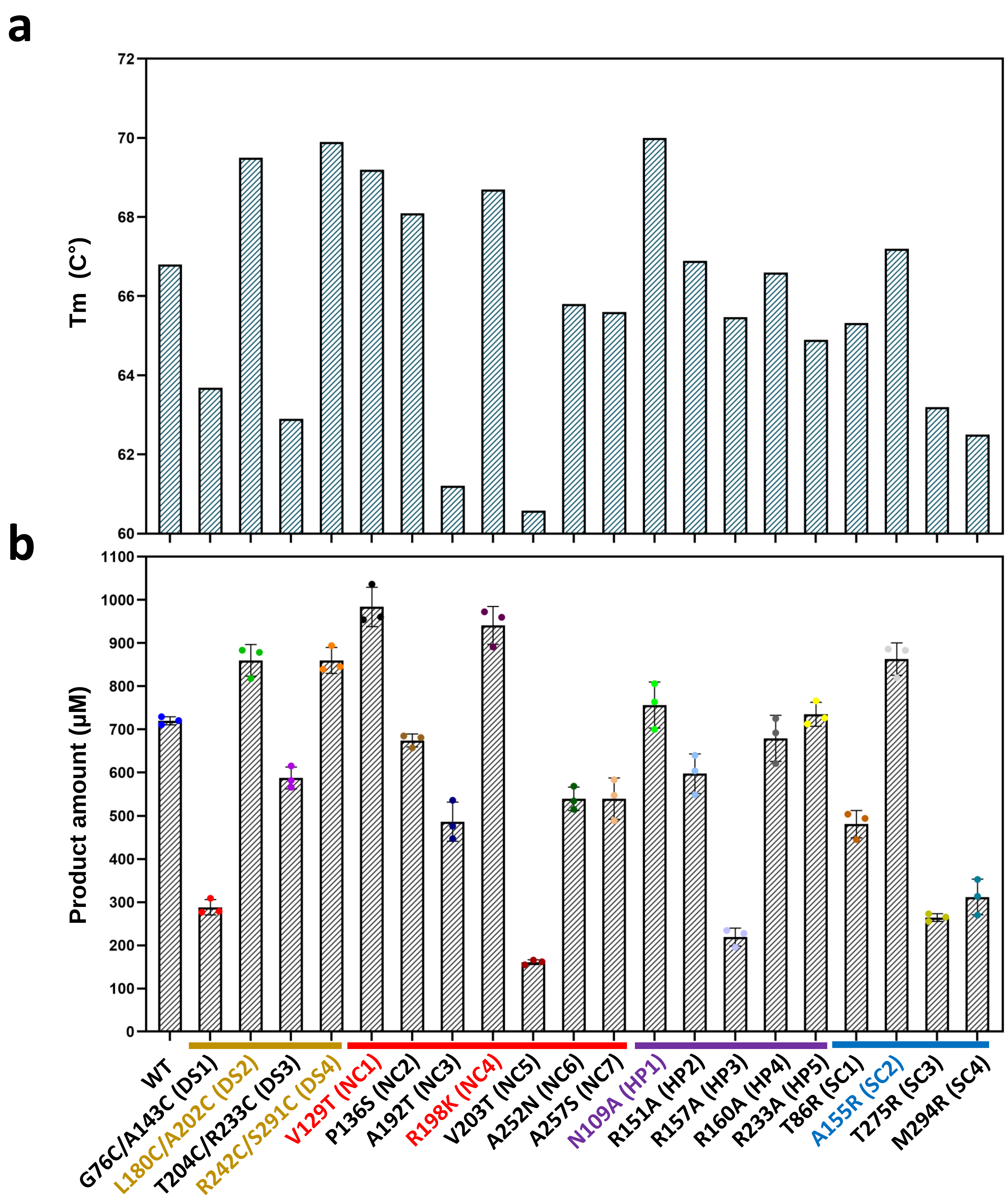

Supplementary Fig. 18. The single-point mutations of *Ca*PETase generated by rational engineering. (a) Thermal stability measurement of the *Ca*PETase variants. (b) PET hydrolytic activities of the *Ca*PETase variants. The reaction was conducted using PC-PET^{Transparent} at 40 °C with 500 nM enzyme in 50 mM Glycine-NaOH pH 9.0 buffer. Reactions were performed in triplicate; Data are presented as mean values ± SD.

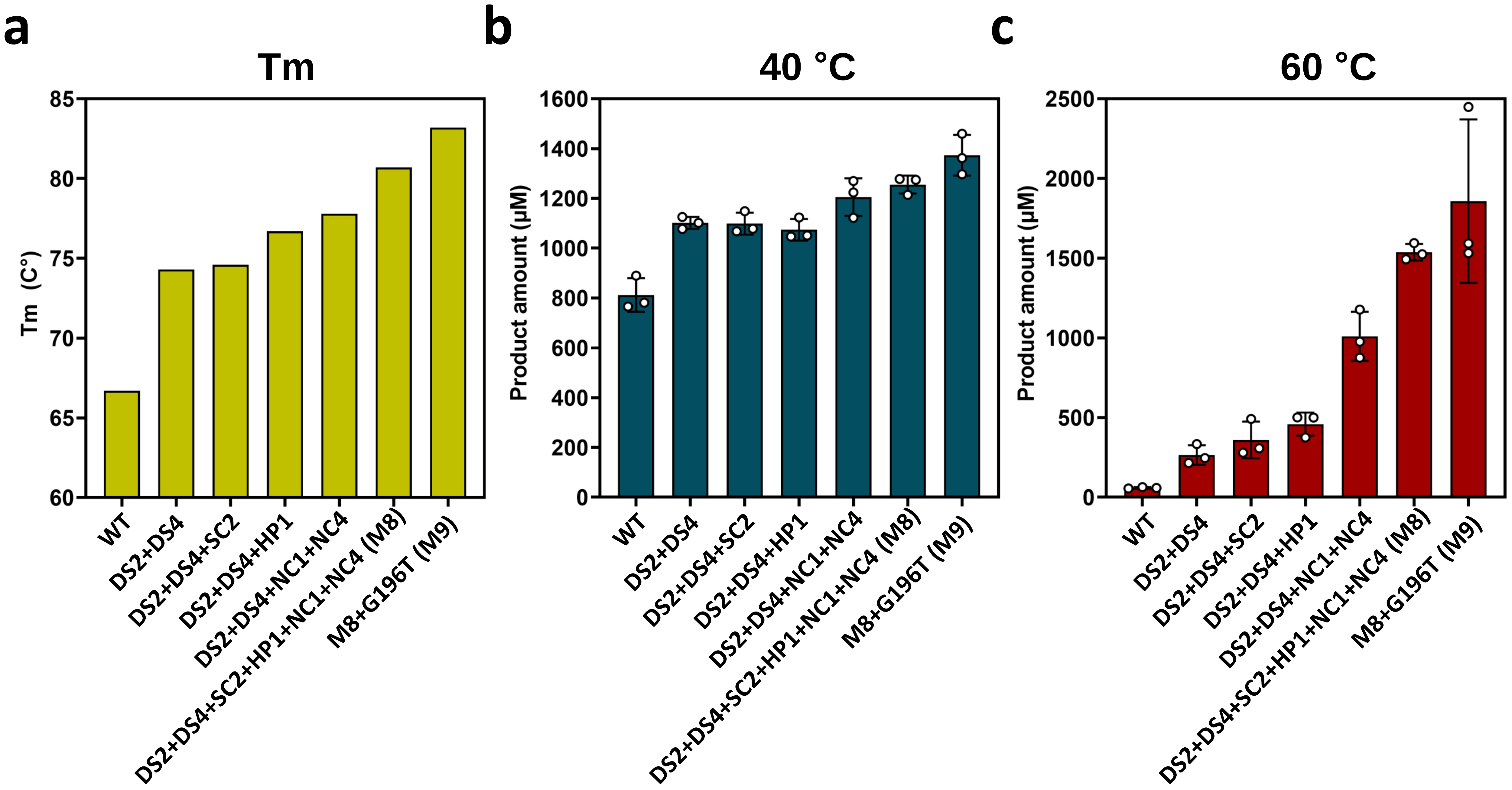

Supplementary Fig. 19. The *Ca*PETase variants generated by combinatorial mutations. (a) Thermal stability measurement of the variants generated by combinatorial strategy. (b,c) PET hydrolytic activity of combinatorial variants. The reaction was conducted using PC-PET^{Transparent} with 500 nM enzyme in 50 mM Glycine-NaOH pH 9.0 buffer. Total amount of TPA, MHET, and BHET released by reacting at 40 °C (b) and 60 °C (c) are shown in the bar chart. Reactions were performed in triplicate; Data are presented as mean values ± SD.

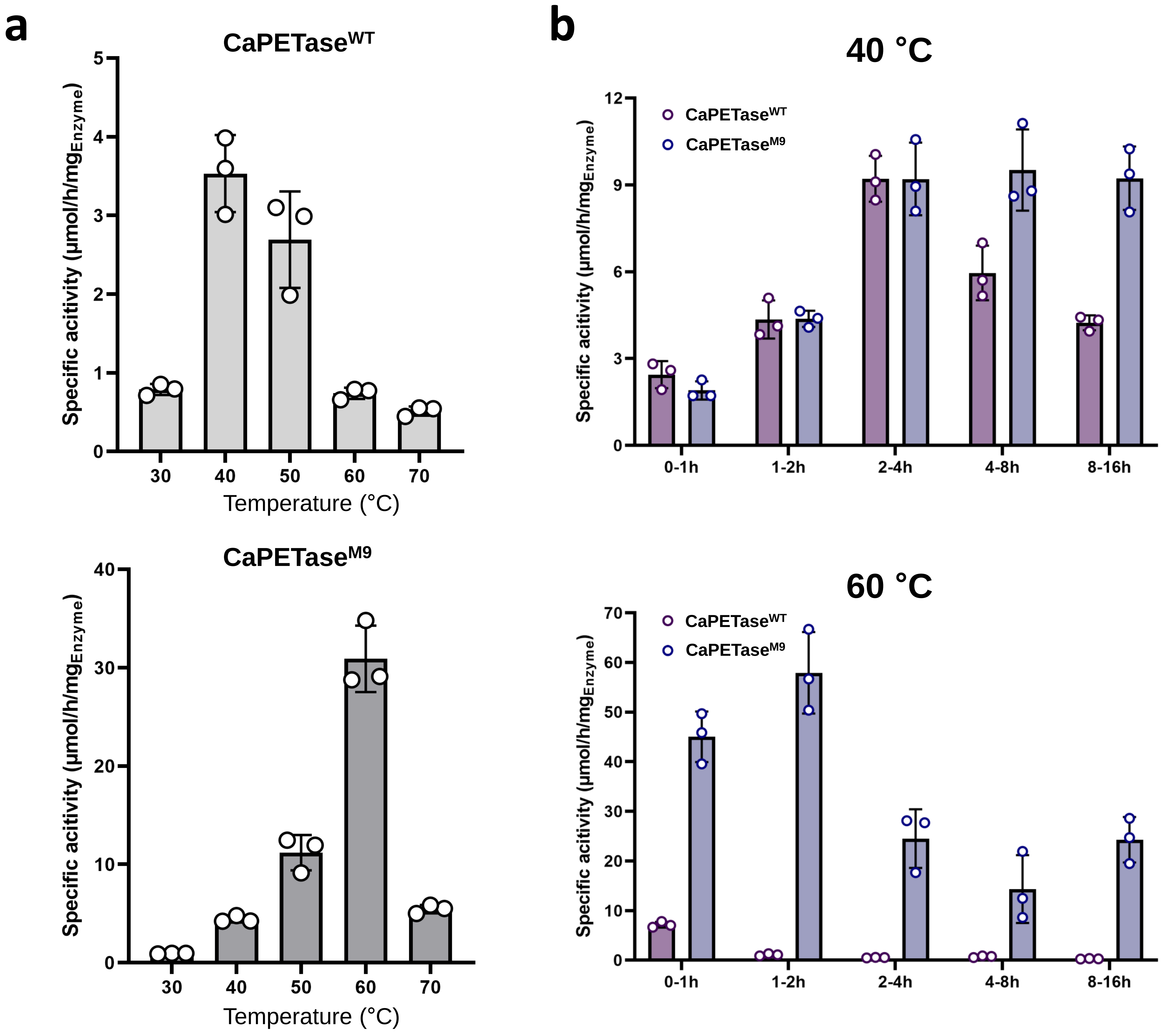

Supplementary Fig. 20. The specific PET hydrolytic activity of *Ca*PETase^{WT} and *Ca*PETase^{M9}. (a) The specific PET hydrolytic activity (Total amount of released TPA, MHET, and BHET within 6 h of reaction) of *Ca*PETase^{WT} and *Ca*PETase^{M9} (1 $mg_{enzyme}g_{PET}^{-1}$) at 30, 40, 50, 60, and 70 °C with PC-PET^{Transparent} in 100 mM Glycine-NaOH (pH 9.0) buffer. The enzyme reactions were carried out in triplicate; error bars represent the s.d. (b) The specific PET hydrolytic activity of *Ca*PETase^{WT} and *Ca*PETase^{M9} at different time points, specially at 40 and 60 °C, with 1 $mg_{enzyme}g_{PET}^{-1}$. The reactions were carried out in triplicate; Reactions were performed in triplicate; Data are presented as mean values ± SD.

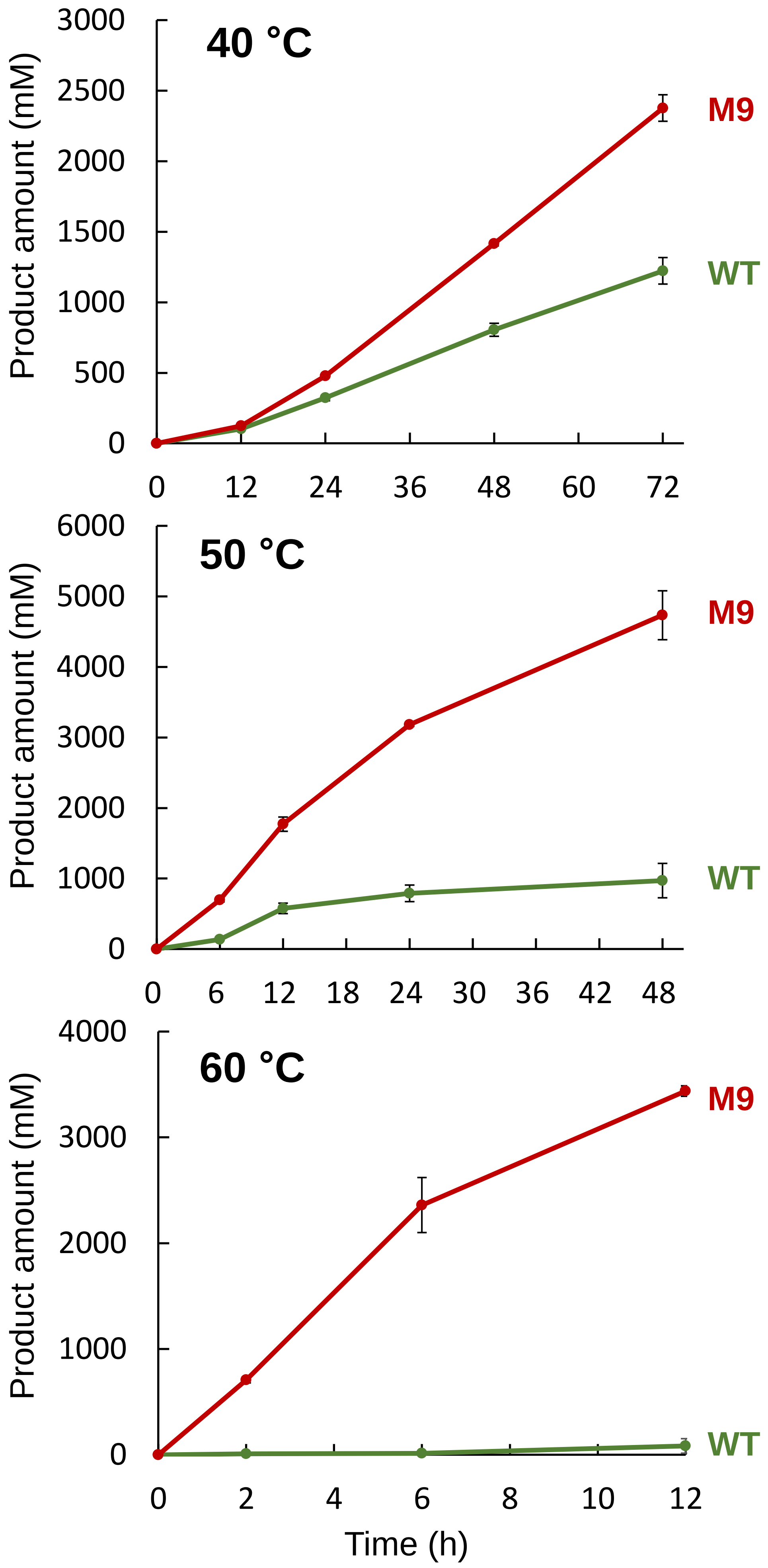

Supplementary Fig. 21. Comparisons of PET hydrolysis activity between *Ca*PETase^{WT} and *Ca*PETase^{M9} at various temperatures on a flask-scale. Comparison of PET hydrolysis activity between *Ca*PETase^{WT} and *Ca*PETase^{M9} at various temperatures on a flask-scale. The reactions were carried out under the 200 mM Glycine-NaOH buffer (pH 9.0) using 1 µM enzyme at 30 ºC, 40 ºC, and 50 ºC with PC-PET (25 mg mL-1) as the substrate. Reactions were performed in triplicate; Data are presented as mean values ± SD.

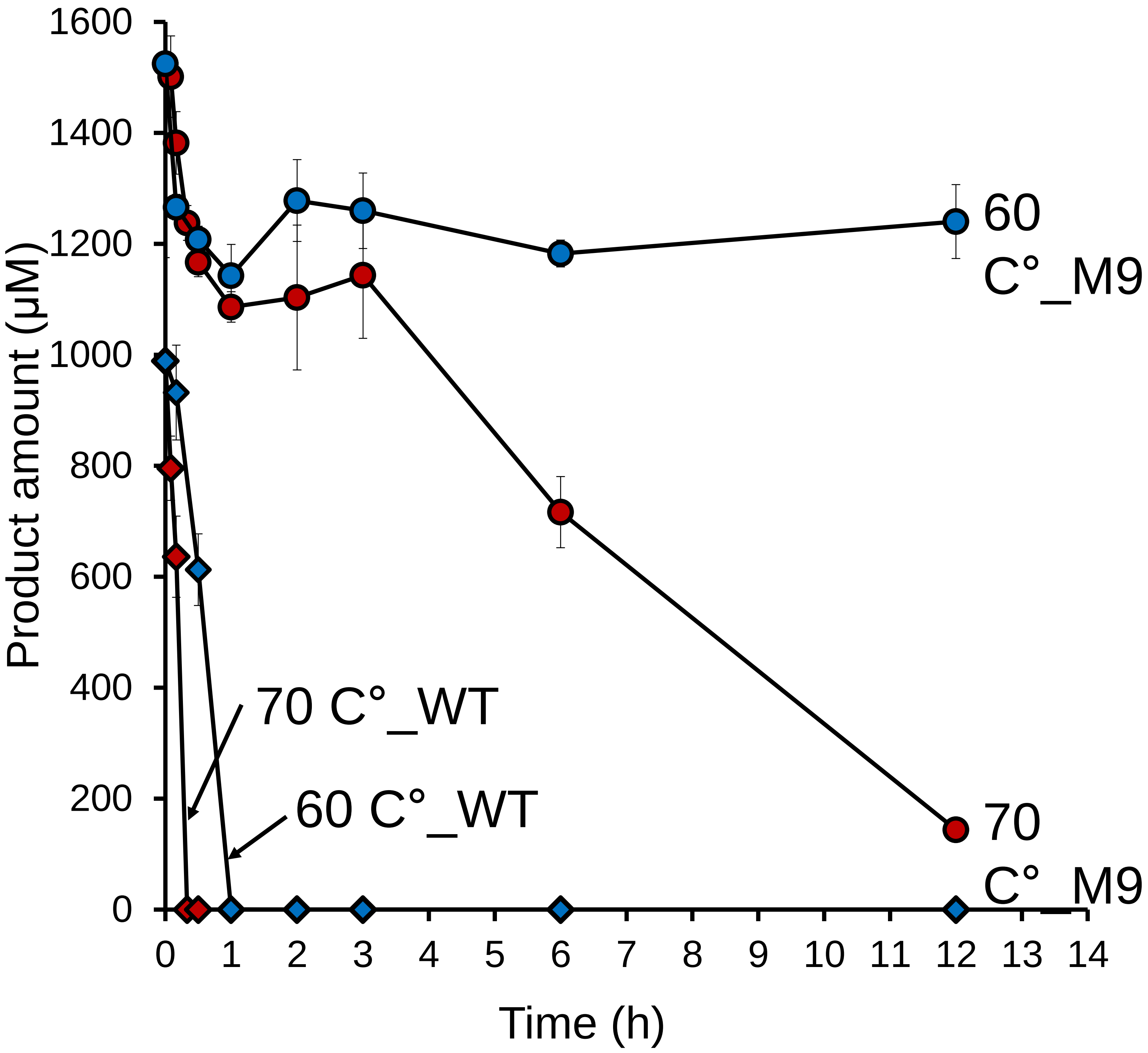

Supplementary Fig. 22. Thermal inactivation of *Ca*PETase^{WT} and *Ca*PETase^{M9}. PET degrading activity was measured using heat-inactivated proteins at 60 and 70 °C for 5 min-12 hrs. Reactions were performed in triplicate; Data are presented as mean values ± SD.

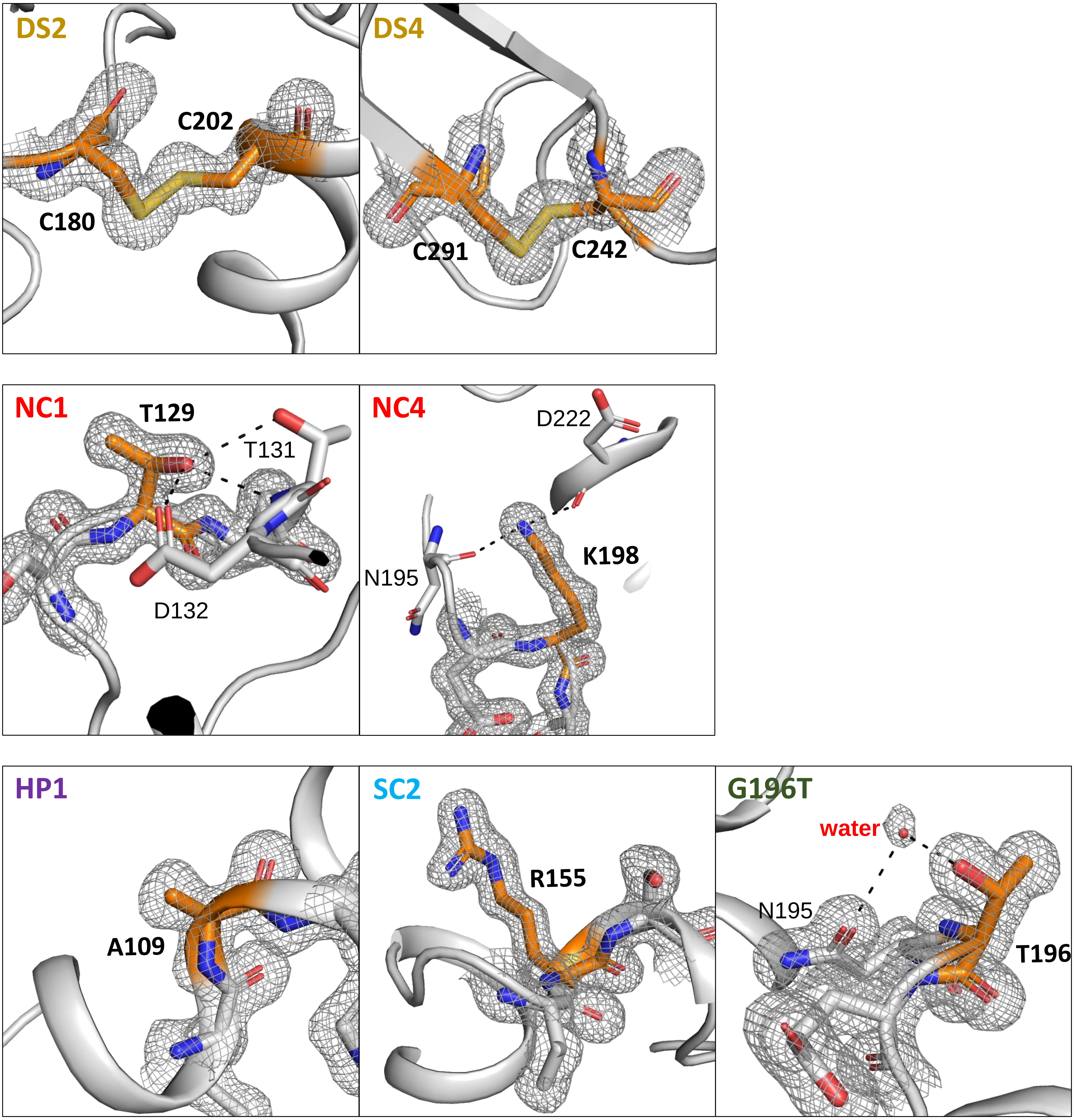

Supplementary Fig. 23. The electron density (Fo-Fc) maps of the introduced mutation points of *Ca*PETase^{M9}. Structures of *Ca*PETase^{M9} are shown as grey-colored cartoon model and mutation points of *Ca*PETase^{M9} are highlighted with orange color as a stick model. Electron density map with 2|Fo| - |Fc| coefficients for mutated residues represented and the map is contoured at 1.0 sigma.

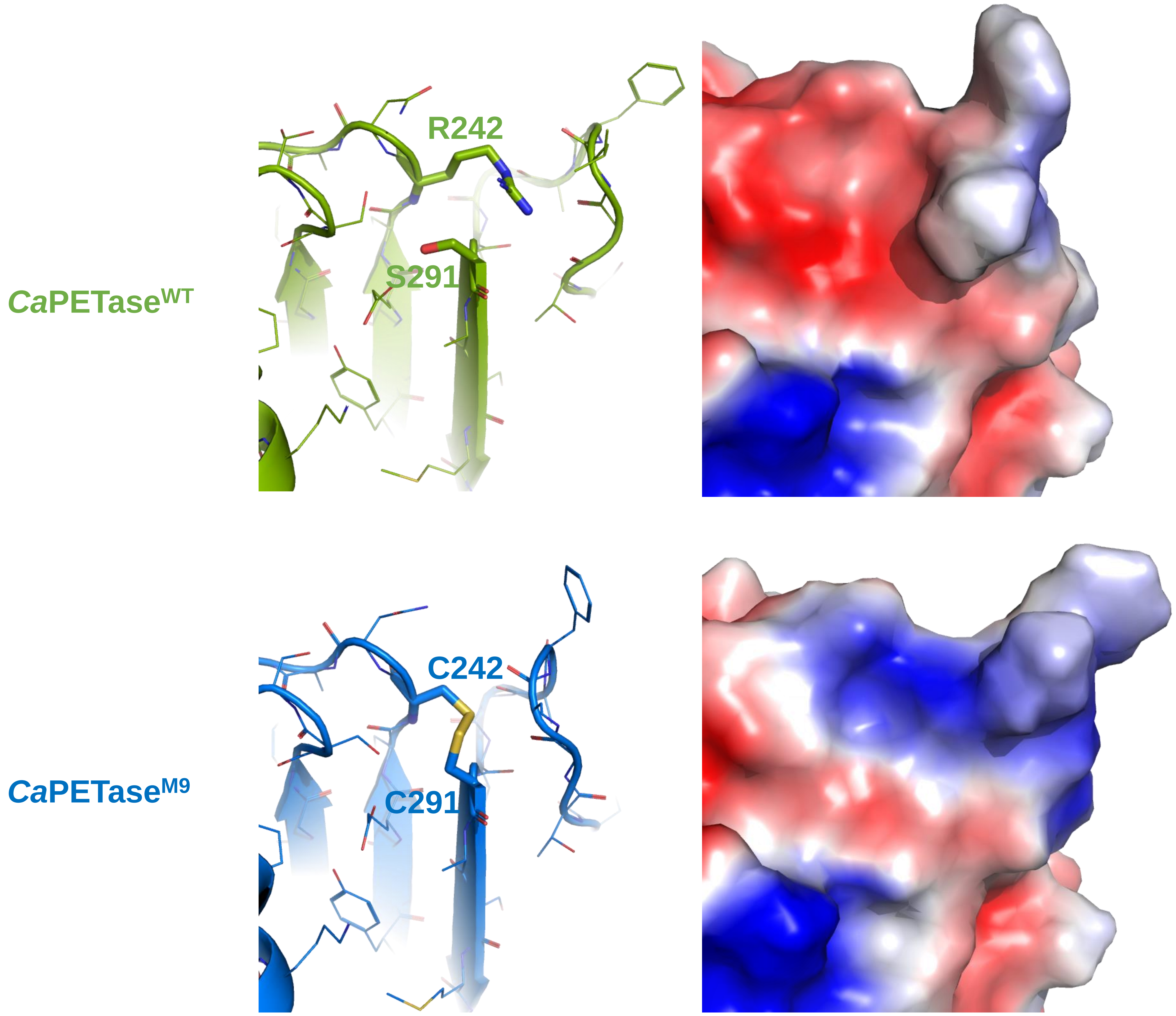

Supplementary Fig. 24. Structural change in the vicinity region of DS4. The DS4 region of *Ca*PETase^{WT} and *Ca*PETase^{M9} are presented as electrostatic potential surface models and cartoon diagrams. The mutation point residues are shown as stick models.

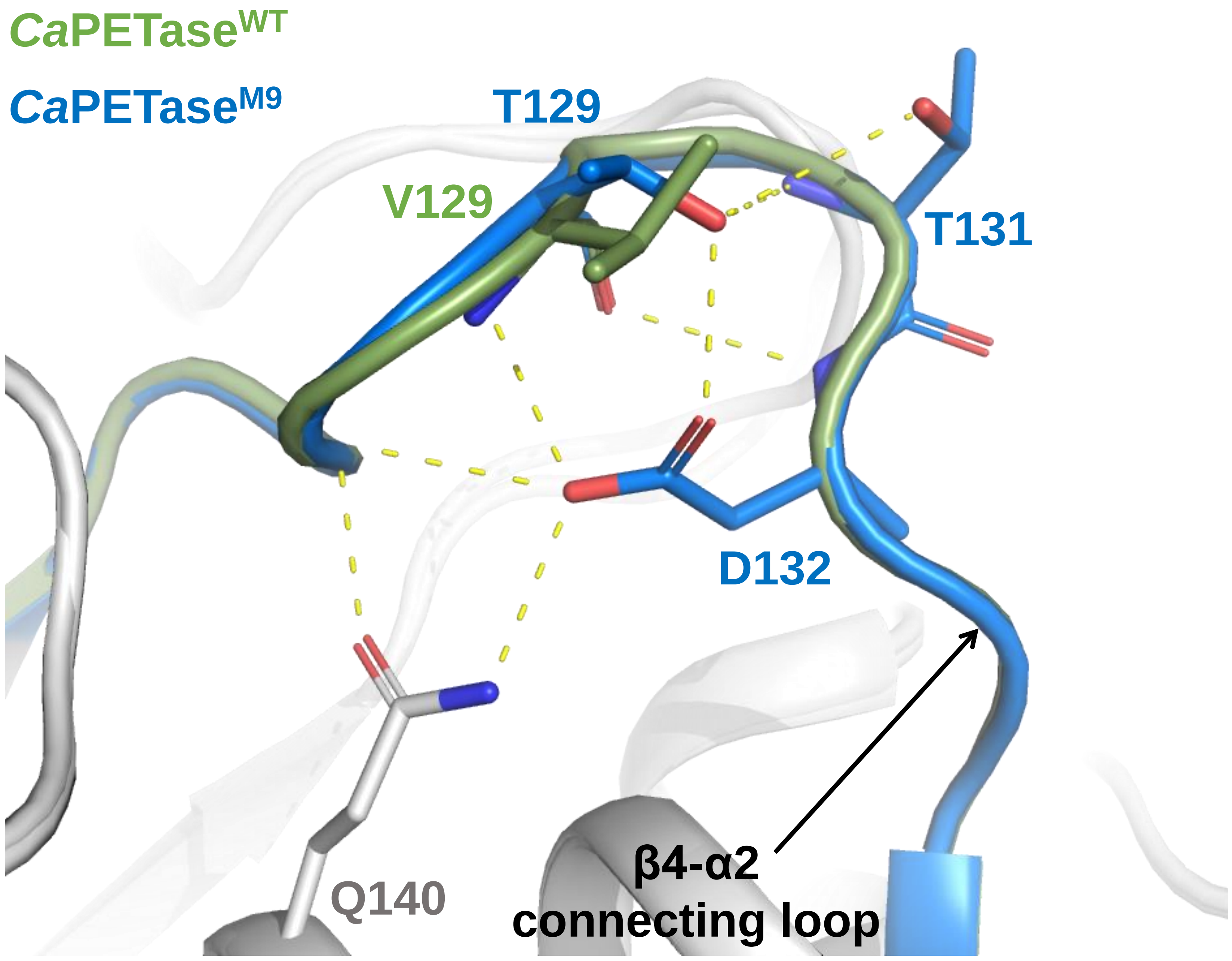

Supplementary Fig. 25. Stabilization mode between V129T and β4-α3 connecting loop. Comparison of β4-α2 connecting loop region between the CaPETase^{WT} and CaPETase^{M9}. The residues involved in stabilization of β4-α2 connecting loop are shown as stick models and the hydrogen bonds involved in the stabilization are presented by yellow-colored dotted-lines.

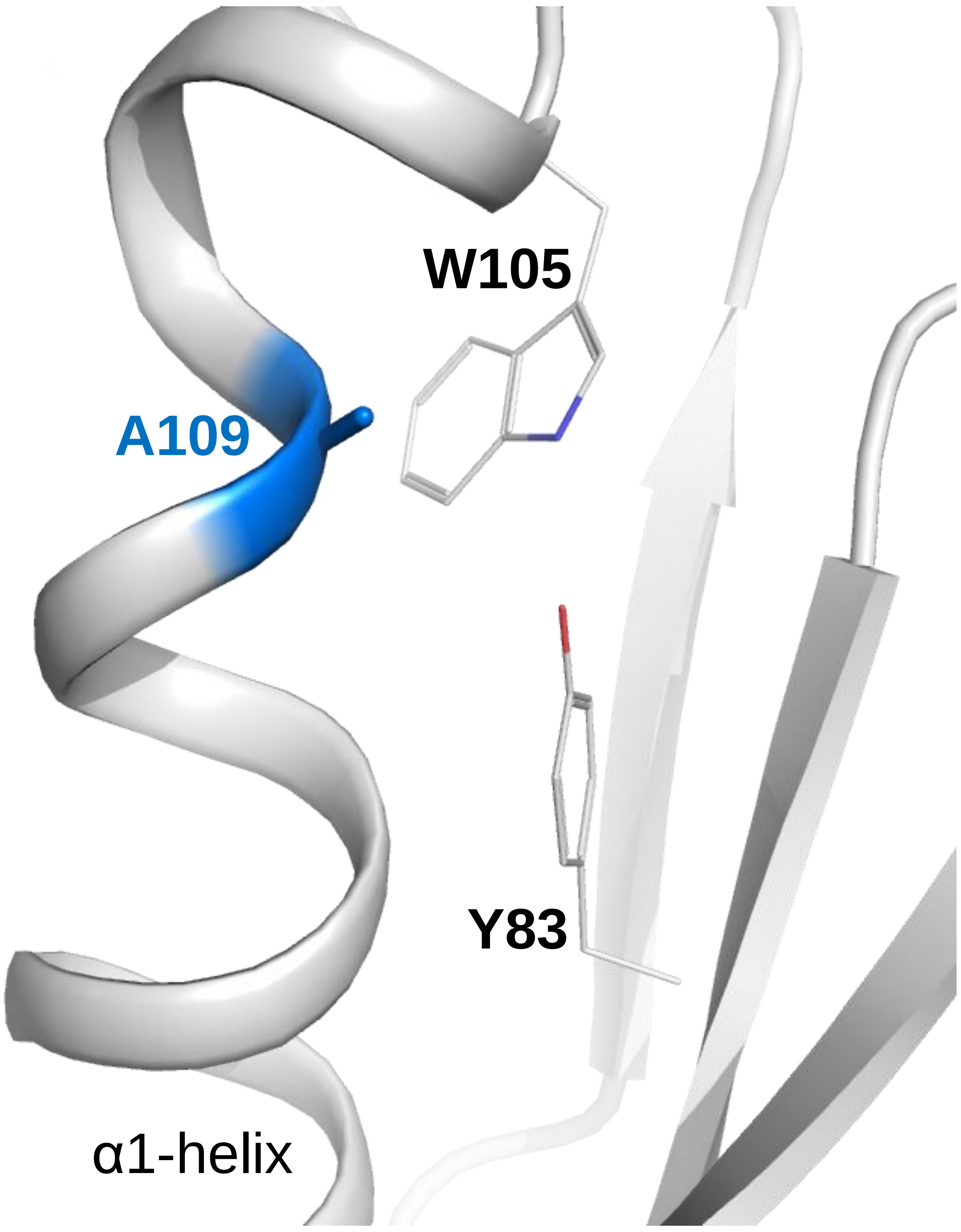

Supplementary Fig. 26. N109A mutations point of CaPETase^{M9}. The structure of CaPETase^{M9} is presented as a cartoon diagram and N109A residue is shown as a stick model. The hydrophobic residues around the N109A are presented as line models.

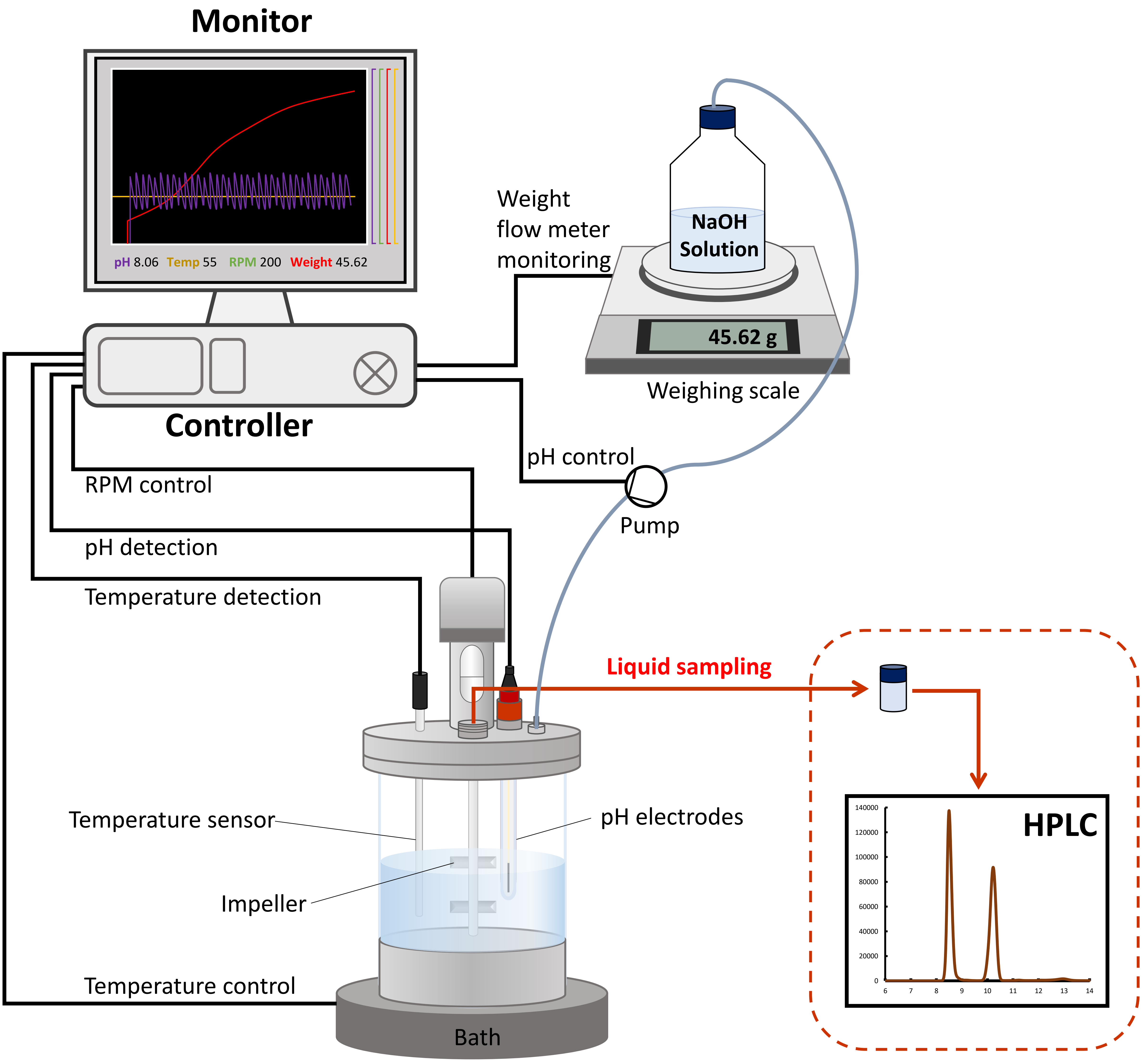

Supplementary Fig. 27. Scheme of the pH-stat bioreactor setup for PET decomposition.

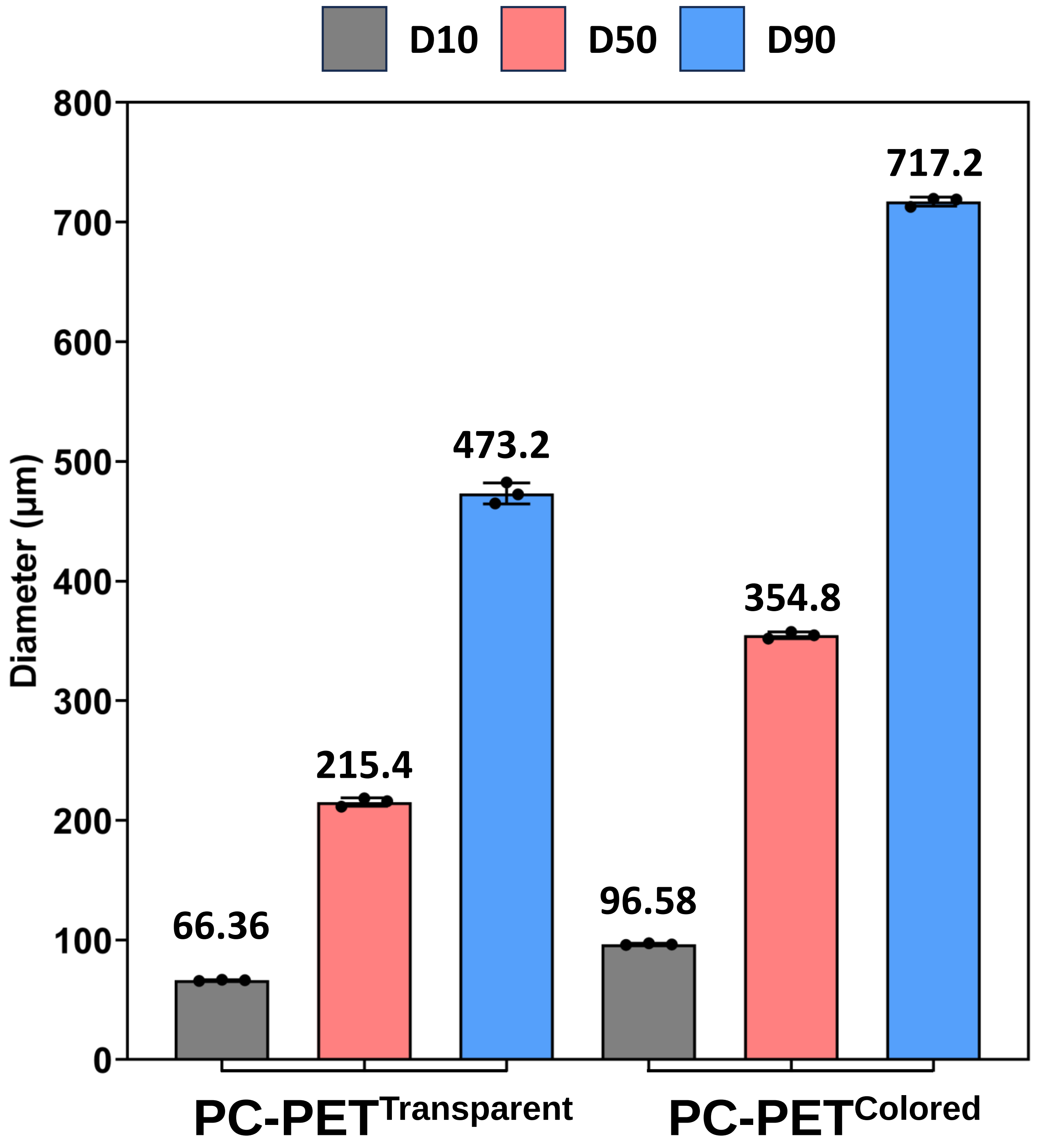

	PC-PET^{Transparent}	PC-PET^{Colored}
**Average Diameter (µm)**	247.8	389.1
**SD**	2.55	2.12

Supplementary Fig. 28. Particle size distribution (D10, D50, and D90) of PC-PET^{Transparent} and PC-PET^{Colored}. Data were collected from Laser Diffraction (LD) analyses. Experiments were carried out in triplicate; Data are presented as mean values ± SD.

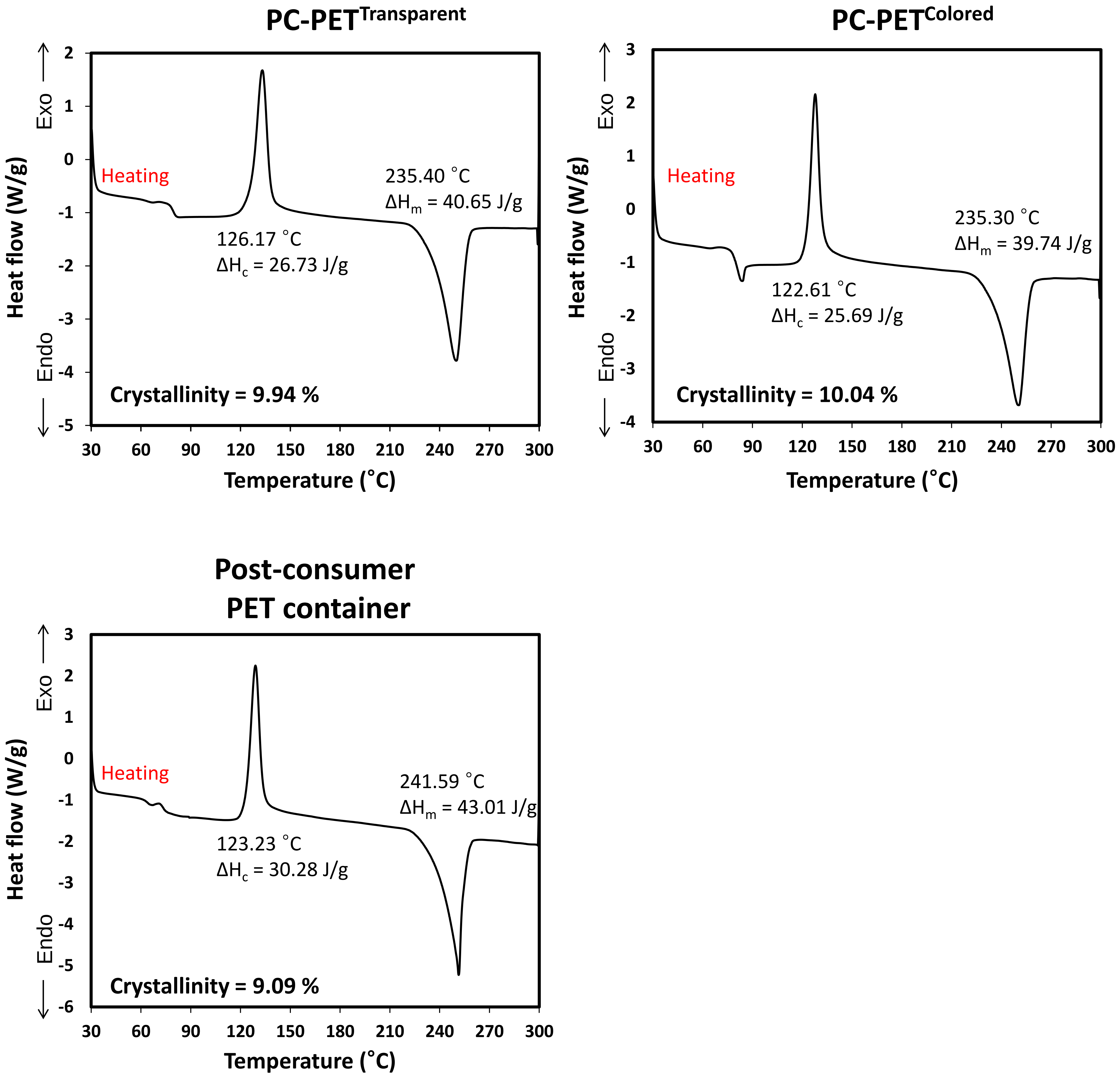

Supplementary Fig. 29. Differential scanning calorimetry (DSC) for crystallinity of PET samples used in this study.

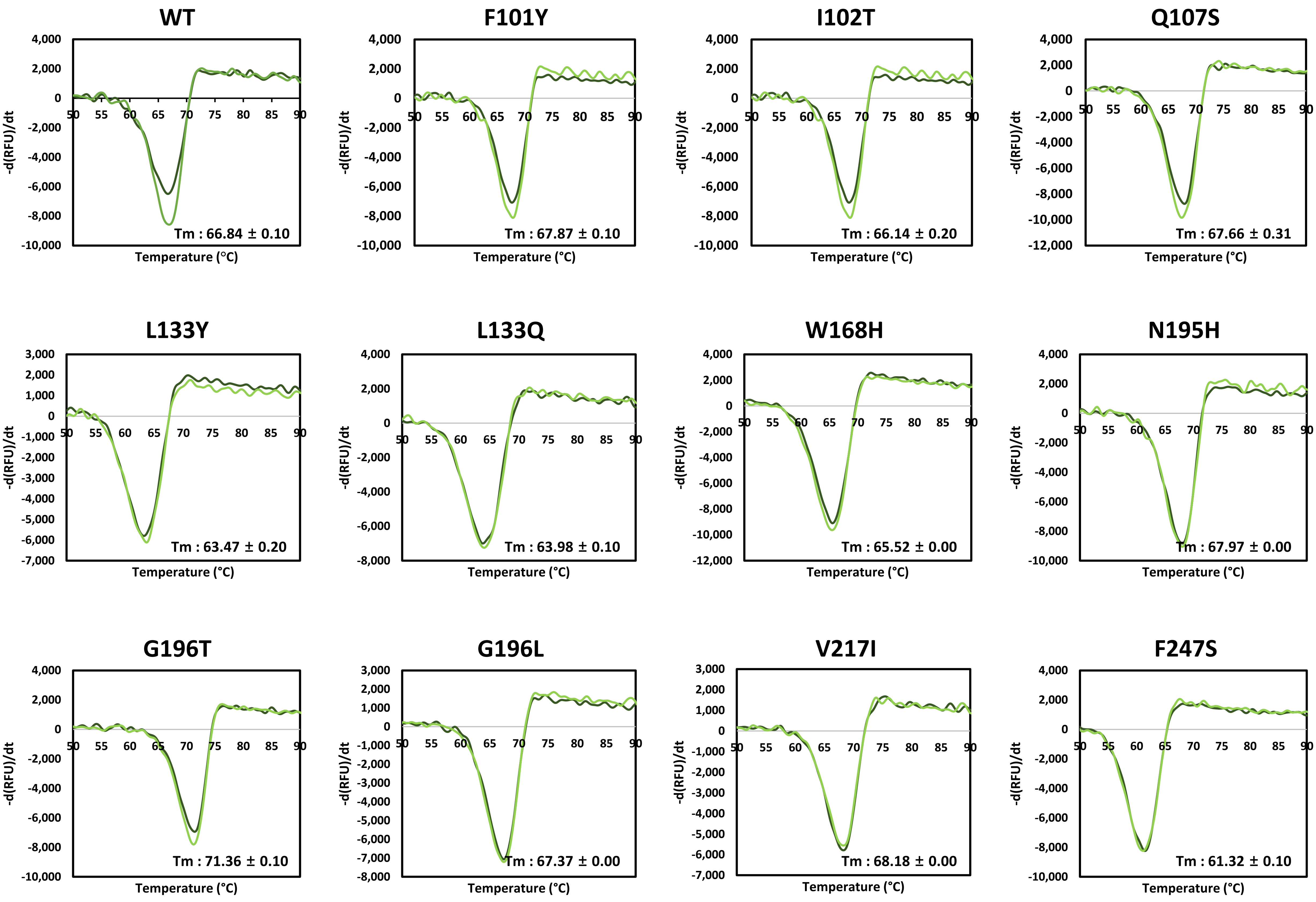

Supplementary Fig. 30. T_m values of wild-type and 39 variants of *Ca*PETase using in this study. Melting curves and melting points of wild-type and 39 variants of *Ca*PETase. Tm values are presented as mean values ± SD, n=2.

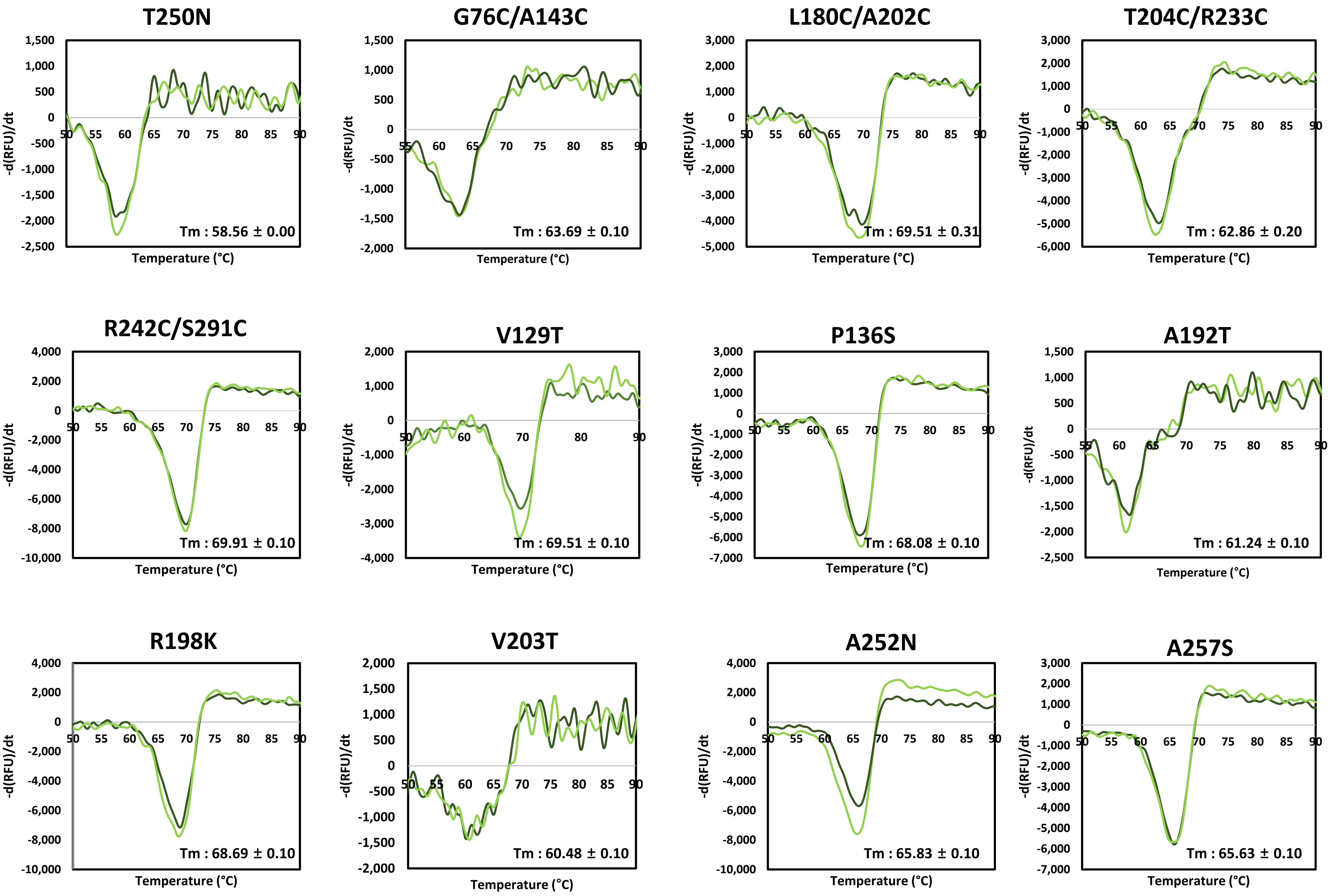

Supplementary Fig. 30. T_m values of wild-type and 39 variants of *Ca*PETase using in this study. (Continued) Melting curves and melting points of wild-type and 39 variants of *Ca*PETase. Tm values are presented as mean values ± SD, n=2.

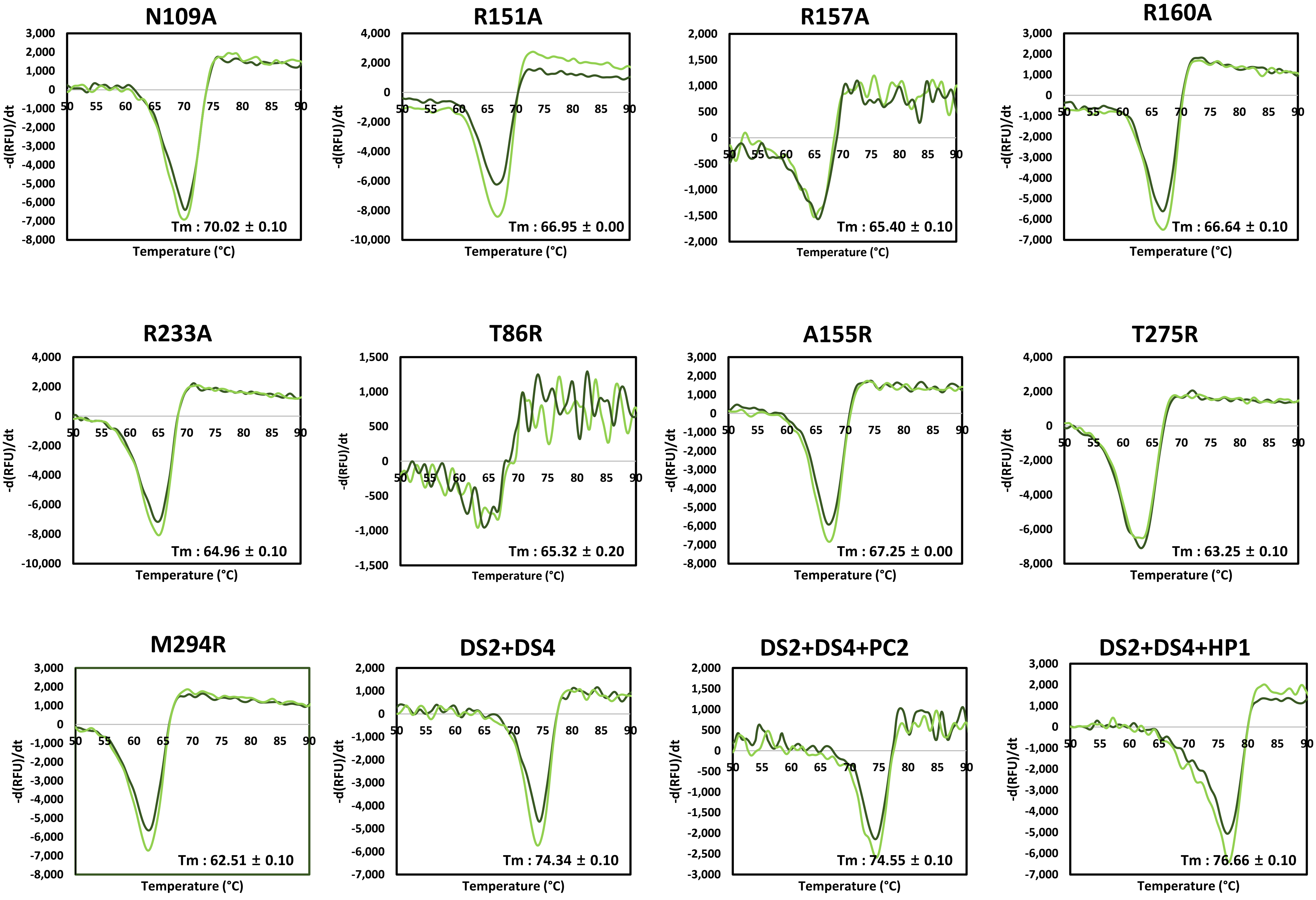

Supplementary Fig. 30. T_m values of wild-type and 39 variants of *Ca*PETase using in this study. (Continued) Melting curves and melting points of wild-type and 39 variants of *Ca*PETase. Tm values are presented as mean values ± SD, n=2.

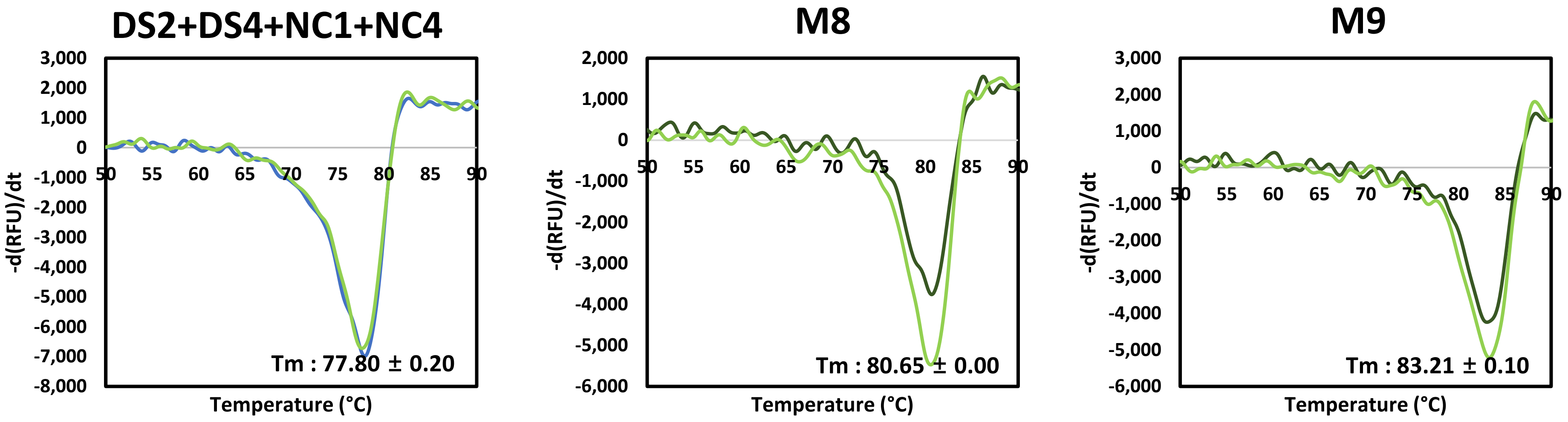

Supplementary Fig. 30. T_m values of wild-type and 39 variants of *Ca*PETase using in this study. (Continued) Melting curves and melting points of wild-type and 39 variants of *Ca*PETase. Tm values are presented as mean values ± SD, n=2.

		1	2	3	4	5	6	7	8	9	10	11	12	13	14	15	16	17	18	19	20	21	22	23	24	25	26	27
1	LCC_AEV21261.1	100	92.15	48.76	45.61	56.54	54.61	56.43	51.03	54.23	52.45	53.63	52.07	52.76	56.03	57.2	45.52	45.88	43.46	41.01	45.91	41.67	45.1	44.32	46.37	47.06	46.82	46.07
2	BhrPETase_GBD22443	92.15	100	47.7	44.21	55.12	53.19	55.71	50.34	52.46	51.75	53.29	52.76	53.45	56.42	57.59	44.09	44.44	42.4	40.29	45.55	40.97	44.41	43.59	44.64	45.59	45.69	44.94
3	KOX11336.1	48.76	47.7	100	47.16	53.15	52.8	54.06	54.51	53.52	53.15	55.21	54.67	55.02	57.14	57.14	45.09	45.82	43.97	38.49	44.29	40.14	44.33	46.35	43.9	48.73	45.02	43.87
4	CaPETase_SHM40309.1	45.61	44.21	47.16	100	58.16	57.3	57.14	51.18	54.17	54.95	51.74	50.85	51.21	54.3	54.69	37.19	38.25	39.1	35.74	40.28	38.62	38.06	45.05	40	43.32	41.82	41.97
5	Tcur_0390_ACY95991.1	56.54	55.12	53.15	58.16	100	81.03	79.17	67.36	65.37	63.29	57.24	57.93	56.9	61.54	61.54	44.4	44.77	44.88	42.96	50.18	43.66	46.1	46.38	44.1	49.1	49.82	47.6
6	WP_103939557.1	54.61	53.19	52.8	57.3	81.03	100	80.62	69.44	63.96	63.29	52.6	53.98	53.98	58.14	58.14	46.01	46.01	44.68	41.88	49.28	46.29	48.4	47.81	46.5	49.09	49.44	46.1
7	Tcur_1278_ACY96861.1	56.43	55.71	54.06	57.14	79.17	80.62	100	69.23	65.71	62.46	56.29	58.04	57.69	62.02	62.02	49.09	49.45	47.33	42.03	51.27	47.87	50.36	48.72	47.02	48.73	51.67	47.96
8	BTA2_WP_131545307.1	51.03	50.34	54.51	51.18	67.36	69.44	69.23	100	71.24	69.28	58.5	58.65	59.32	63.95	64.34	45.21	46.92	42.42	41.26	46.74	43.96	44.93	49.64	44.48	48.42	49.64	48.01
9	WP_125313231.1	54.23	52.46	53.52	54.17	65.37	63.96	65.71	71.24	100	78.74	57.64	57.95	60.21	63.95	65.12	47.54	48.24	43.94	41.49	46.48	44.48	44.79	50	48.14	52.19	49.44	48.52
10	WP_075975245.1	52.45	51.75	53.15	54.95	63.29	63.29	62.46	69.28	78.74	100	56.85	58	58.36	63.18	62.79	46.02	48.1	42.03	40.42	46.71	42.57	43.54	48.2	46.82	49.64	50	48.91
11	WP_068752972.1	53.63	53.29	55.21	51.74	57.24	52.6	56.29	58.5	57.64	56.85	100	84	81.67	90.08	85.5	43.66	47.54	45.33	43.62	47.02	42.81	46.9	46.26	45.24	50.54	49.45	48.35
12	TfCut1_CBY05529.1	52.07	52.76	54.67	50.85	57.93	53.98	58.04	58.65	57.95	58	84	100	95.02	96.95	93.89	42.61	45.7	45.12	44.6	46.92	42.14	46.13	47.18	45.13	49.65	49.64	49.64
13	TfCut2_E5BBQ3	52.76	53.45	55.02	51.21	56.9	53.98	57.69	59.32	60.21	58.36	81.67	95.02	100	92.75	99.62	44.01	47.18	47.24	45.23	48.25	43.69	46.74	47.69	47.46	50.9	49.82	50.55
14	Thc_Cut2_E9LVH9	56.03	56.42	57.14	54.3	61.54	58.14	62.02	63.95	63.95	63.18	90.08	96.95	92.75	100	93.13	46.51	50	49.03	47.86	52.14	47.29	52.33	48.84	51.34	51.74	50.97	51.78
15	Thc_Cut1_E9LVH8	57.2	57.59	57.14	54.69	61.54	58.14	62.02	64.34	65.12	62.79	85.5	93.89	99.62	93.13	100	46.9	50.39	49.81	49.03	52.92	48.45	51.55	50	52.87	53.67	51.75	52.96
16	WP_083724990.1	45.52	44.09	45.09	37.19	44.4	46.01	49.09	45.21	47.54	46.02	43.66	42.61	44.01	46.51	46.9	100	94.08	60.33	48.97	57.77	53.16	57.48	44.13	47.33	47.72	47.67	45.2
17	PE-H_A0A1H6AD45	45.88	44.44	45.82	38.25	44.77	46.01	49.45	46.92	48.24	48.1	47.54	45.7	47.18	50	50.39	94.08	100	59.33	49.66	57.09	53.49	57.14	45.91	48.67	49.47	49.82	47.69
18	LipIAF5-2_PET2_C3RYL0	43.46	42.4	43.97	39.1	44.88	44.68	47.33	42.42	43.94	42.03	45.33	45.12	47.24	49.03	49.81	60.33	59.33	100	52.72	60.74	55.26	59.87	47.89	52.46	49.66	49.65	48.25
19	PET6_SHF85073.1	41.01	40.29	38.49	35.74	42.96	41.88	42.03	41.26	41.49	40.42	43.62	44.6	45.23	47.86	49.03	48.97	49.66	52.72	100	62.54	52.03	53.74	41.16	46.92	49.29	49.26	45.99
20	MBA55398.1	45.91	45.55	44.29	40.28	50.18	49.28	51.27	46.74	46.48	46.71	47.02	46.92	48.25	52.14	52.92	57.77	57.09	60.74	62.54	100	60.73	65.89	44.09	51.68	52.82	51.8	47.84
21	PET5_R4YKL9	41.67	40.97	40.14	38.62	43.66	46.29	47.87	43.96	44.48	42.57	42.81	42.14	43.69	47.29	48.45	53.16	53.49	55.26	52.03	60.73	100	72.4	44.01	52.63	50.87	51.96	49.47
22	MAM88718.1	45.1	44.41	44.33	38.06	46.1	48.4	50.36	44.93	44.79	43.54	46.9	46.13	46.74	52.33	51.55	57.48	57.14	59.87	53.74	65.89	72.4	100	44.72	54.93	51.56	52.67	50.18
23	RZL00883.1	44.32	43.59	46.35	45.05	46.38	47.81	48.72	49.64	50	48.2	46.26	47.18	47.69	48.84	50	44.13	45.91	47.89	41.16	44.09	44.01	44.72	100	51.54	54.42	54.51	50.54
24	BbPETase_A0A1F4JXW8	46.37	44.64	43.9	40	44.1	46.5	47.02	44.48	48.14	46.82	45.24	45.13	47.46	51.34	52.87	47.33	48.67	52.46	46.92	51.68	52.63	54.93	51.54	100	64.77	62.67	60
25	PET12_A0A0G3BI90	47.06	45.59	48.73	43.32	49.1	49.09	48.73	48.42	52.19	49.64	50.54	49.65	50.9	51.74	53.67	47.72	49.47	49.66	49.29	52.82	50.87	51.56	54.42	64.77	100	65.86	63.54
26	RgPETase_A0A1W6L588	46.82	45.69	45.02	41.82	49.82	49.44	51.67	49.64	49.44	50	49.45	49.64	49.82	50.97	51.75	47.67	49.82	49.65	49.26	51.8	51.96	52.67	54.51	62.67	65.86	100	75.27
27	IsPETase_GAP38373.1	46.07	44.94	43.87	41.97	47.6	46.1	47.96	48.01	48.52	48.91	48.35	49.64	50.55	51.78	52.96	45.2	47.69	48.25	45.99	47.84	49.47	50.18	50.54	60	63.54	75.27	100

Supplementary Fig. 31. Percent identity values for sequences used in phylogenetic analysis.

Supplementary Table 1. List of enzymes used for phylogenetic tree analysis.

Enzyme	Accession code	Organism	Length	Ref.
*Ca*PETase	SHM40309.1	*Cryptosporangium aurantiacum*	299	-
-	WP_083724990.1	*Halopseudomonas pachastrellae*	304	-
-	MBA55398.1	*Pseudomonadales* bacterium	303	-
-	MAM88718.1	*Hahellaceae* bacterium (marine metagenome)	308	-
-	RZL00883.1	*Rubrivivax* sp.	295	-
-	WP_103939557.1	*Thermomonospora echinospora*	292	-
-	WP_125313231.1	*Amycolatopsis eburnean*	309	-
-	WP_075975245.1	*Actinokineospora bangkokensis*	307	-
-	WP_068752972.1	*Thermobifida cellulosilytica*	300	-
-	KOX11336.1	*Nocardiopsis* sp. NRRL B-16309	292	-
*Tf*Cut1	CBY05529.1	*Thermobifida fusca*	319	1
*Tf*Cut2	E5BBQ3	*Thermobifida fusca*	301	1
*Thc*_Cut1	E9LVH9	*Thermobifida cellulosilytica*	262	2
*Thc*_Cut2	ADV92527.1	*Thermobifida cellulosilytica*	262	2
LCC	AEV21261.1	uncultured bacterium	293	3
*Bhr*PETase	GBD22443	bacterium HR29	293	4
PE-H	WP_088276085.1	*Halopseudomonas aestusnigri*	304	5
PET5	R4YKL9	*Oleispira antarctica* RB-8	310	6
PET2	ACC95208.1	uncultured bacterium	308	6
*Bb*PETase	OGB27210.1	*Burkholderiales* bacterium RIFCSPLOWO2_02_FULL_57_36	426	7
PET12	WP_047194864.1	*Schlegelella brevitalea*	298	6
*Rg*PETase	WP_085749752.1	*Rhizobacter gummiphilus*	292	8
*Is*PETase	GAP38373.1	*Ideonella sakaiensis*	290	9
*Tcur*_1278	ACY96861.1	*Thermomonospora curvata* DSM 43183	289	10
*Tcur*_0390	ACY95991.1	*Thermomonospora curvata* DSM 43183	292	10
BTA2	WP_131545307.1	unclassified *Streptomyces*	320	11

Supplementary Table 2. Data collection and refinement statistics of *Ca*PETase and *Ca*PETase^{M9}.

	***Ca*PETase**	***Ca*PETase^{M9}**
**PDB code**	7YM9	7YME
**Data collection**		
Wavelength (Å)	0.97934	0.97934
Unit cell (a, b, c; α, β, γ) (Å; °)	82.309, 82.309, 87.391; 90.0, 90.0, 90.0	41.019, 112.31, 112.54; 90.0, 90.0, 90.0
Space group	$P2_12_12$	$P2_12_12$
Solvent content (%)	52.25	45.37
Protein chains in AU	2	2
Resolution range (Å)	26.23-1.34	39.78-1.50
Highest resolution shell (Å)	1.36-1.34	1.53-1.50
Unique reflections	133696	84238
Redundancy	12.4(10.4)	7.2(7.2)
Completeness (%)	99.7(99.8)	100.0(100.0)
R_{merge} (%)[a]	8.0(51.2)	8.0(82.9)
CC(1/2)	0.998(0.948)	0.996(0.779)
Average I/σ(I)	52.7(4.4)	31.27(2.47)
**Refinement**		
R (%)[b]	17.2	17.6
R_{free} (%)[c]	18.3	20.2
Mean B value (Å^2)	13.8	17.1
RMS deviation bond lengths (Å)	0.017	0.011
RMS deviation bond angles (°)	2.001	1.750
Number of amino acid residues	514	518
Number of water molecules	470	453
Ramachandran plot		
Favored regions (%)	95.10	95.50
Allowed regions (%)	4.49	4.11
Outlier regions (%)	0.41	0.39
Clash score	1.92	2.07

Note: Data collection and processing values for the outer shell was given in parentheses.

[a] Rmerge = Σ|I – <I>|/ΣI, where I is the integrated intensity of a given reflection.

[b] R = Σ||*Fobs*| - |*Fcalc*||/Σ| *Fobs*|. [c] R_{free} was calculated using 5% of data excluded from refinement.

Supplementary Table 3. Molecular weights (M_n, M_w, M_p) of PC-PET^{Transparent} and PC-PET^{Colored} determined by gel permeation chromatography (GPC) analysis.

Substrate	Molecular Weight			
	*M*n[a]	*M*w[b]	*M*p[c]	*M*w/*M*n[d]
**PC-PET^{Transparent}**	7060	16700	13400	2.37
**PC-PET^{Colored}**	6820	17900	14600	2.63

[a] *M*n: Number-average molecular weight
[b] *M*w: Weight-average molecular weight
[c] *M*p: Molecular weight of the highest peak
[d] *M*w/*M*n: Polydispersity index

Supplementary Table 4. Primer lists used in this study.

Enzyme	Forward primer (5'-3')	Reverse primer (5'-3')
*Ca*PETase^{WT}	TATATCATATGGCGGCCGACAACCCCTACCAAC	TATATCTCGAGGAACGGGCAGGTGTTCATCGAC
*Ca*PETase^{I102T}	GATCGTGCCGGGCTTCACCTCGGTCTGGGCCC	GGGCCCAGACCGAGGTGAAGCCCGGCACGATC
*Ca*PETase^{Q107S}	ATCTCGGTCTGGGCCAGTCTCAACTGGCTCGGG	CCCGAGCCAGTTGAGACTGGCCCAGACCGAGAT
*Ca*PETase^{L133Y}	CAGCGTGATCACCGACTACCCCGACCCGCGCGG	CCGCGCGGGTCGGGGTAGTCGGTGATCACGCTG
*Ca*PETase^{L133Q}	CAGCGTGATCACCGACCAACCCGACCCGCGCGG	CCGCGCGGGTCGGGTTGGTCGGTGATCACGCTG
*Ca*PETase^{W168H}	CTGGCCGCGGCCGGCCACTCGATGGGCGGCGGC	GCCGCCGCCCATCGAGTGGCCGGCCGCGGCCAG
*Ca*PETase^{N195H}	GGGATGGCGCCCTGGCACGGCGAGCGCAACTGG	CCAGTTGCGCTCGCCGTGCCAGGGCGCCATCCC
*Ca*PETase^{G196T}	GATGGCGCCCTGGAACACCGAGCGCAACTGGTC	GACCAGTTGCGCTCGGTGTTCCAGGGCGCCATC
*Ca*PETase^{G196L}	GATGGCGCCCTGGAACCTCGAGCGCAACTGGTC	GACCAGTTGCGCTCGAGGTTCCAGGGCGCCATC
*Ca*PETase^{T250N}	GACCATTTCTTCCCCAATTCGGCGAACACCACG	CGTGGTGTTCGCCGAATTGGGGAAGAAATGGTC
*Ca*PETase^{G76C}	ATCGTCGGTGCCTCCTGCTTCGGCGGCGGCCAG	CTGGCCGCCGCCGAAGCAGGAGGCACCGACGAT
*Ca*PETase^{A143C}	GGTGACCAGGCGCTGTGCGCGCTGGACTGGGCG	CGCCCAGTCCAGCGCGCACAGCGCCTGGTCACC
*Ca*PETase^{L180C}	CTGCGGCGGGCCGCATGTCAACGCCCGTCGCTC	GAGCGACGGGCGTTGACATGCGGCCCGCCGCAG
*Ca*PETase^{A202C}	GAGAAGAACTGGTCCTGCGTCACCGTGCCGACG	CGTCGGCACGGTGACGCAGGACCAGTTCTTCTC
*Ca*PETase^{T204C}	AACTGGTCCGCCGTCTGCGTGCCGACGCTGTTC	GAACAGCGTCGGCACGCAGACGGCGGACCAGTT
*Ca*PETase^{R233C}	CTACAACAGCATCACCTGCGCCGAGAAGGATTAC	GTAATCCTTCTCGGCGCAGGTGATGCTGTTGTAG

*underlined regions indicating NdeI and XhoI restriction sites

Supplementary Table 4. Primer lists used in this study.

Enzyme	Forward primer (5’-3’)	Reverse primer (5’-3’)
*Ca*PETase^{R242C}	GATTACATCGAACTCTGCAACGCCGACCATTTC	GAAATGGTCGGCGTTGCAGAGTTCGATGTAATC
*Ca*PETase^{S291C}	TTGTTCGCCCCGGTGTGCGCGTCGATGAACACC	GGTGTTCATCGACGCGCACACCGGGGCGAACAA
*Ca*PETase^{V129T}	GGCATCGAGACCAGCACGATCACCGACCTGCCC	GGGCAGGTCGGTGATCGTGCTGGTCTCGATGCC
*Ca*PETase^{P136S}	ACCGACCTGCCCGACTCGCGCGGTGACCAGGCG	CGCCTGGTCACCGCGCGAGTCGGGCAGGTCGGT
*Ca*PETase^{A192T}	GCGATCGTCGGGATGACGCCCTGGAACGGCGAG	CTCGCCGTTCCAGGGCGTCATCCCGACGATCGC
*Ca*PETase^{R198K}	CCCTGGAACGGCGAGAAGAACTGGTCCGCCGTC	GACGGCGGACCAGTTCTTCTCGCCGTTCCAGGG
*Ca*PETase^{V203T}	CGCAACTGGTCCGCCACCACCGTGCCGACGCTG	CAGCGTCGGCACGGTGGTGGCGGACCAGTTGCG
*Ca*PETase^{A252N}	TTCTTCCCCACGTCGAACAACACCACGATGGCG	CGCCATCGTGGTGTTGTTCGACGTGGGGAAGAA
*Ca*PETase^{A257S}	GCGAACACCACGATGTCGAAATACTTCATCTCG	CGAGATGAAGTATTTCGACATCGTGGTGTTCGC
*Ca*PETase^{N109A}	GTCTGGGCCCAGCTCGCCTGGCTCGGGCCGCGC	GCGCGGCCCGAGCCAGGCGAGCTGGGCCCAGAC
*Ca*PETase^{R151A}	GACTGGGCGACGACCGCGAGCCCGGTCGCGAGC	GCTCGCGACCGGGCTCGCGGTCGTCGCCCAGTC
*Ca*PETase^{R157A}	AGCCCGGTCGCGAGCGCGATCGACCGACCCGG	CCGGGTCCGGTCGATCGCGCTCGCGACCGGGCT
*Ca*PETase^{R160A}	GCGAGCCGGATCGACGCGACCCGGCTGGCCGCG	CGCGGCCAGCCGGGTCGCGTCGATCCGGCTCGC
*Ca*PETase^{R233A}	TACAACAGCATCACCGCAGCCGAGAAGGATTAC	GTAATCCTTCTCGGCTGCGGTGATGCTGTTGTA
*Ca*PETase^{T86R}	CAGATCTACTACCCGAGGGACACCAGCCAGACG	CGTCTGGCTGGTGTCCCTCGGGTAGTAGATCTG
*Ca*PETase^{A155R}	ACCCGGAGCCCGGTCCGGAGCCGGATCGACCGG	CCGGTCGATCCGGCTCCGGACCGGGCTCCGGGT

Supplementary Table 4. Primer lists used in this study.

Enzyme	Forward primer (5'-3')	Reverse primer (5'-3')
*Ca*PETase^{T275R}	AACGACACCCGCTACCGCCAGTTCCTCTGCCCC	GGGGCAGAGGAACTGGCGGTAGCGGGTGTCGTT
*Ca*PETase^{M294R}	CCGGTGTGCGCGTCGCGGAACACCTGCCCGTTC	GAACGGGCAGGTGTTCCGCGACGCGCACACCGG

Supplementary References

1. Then, J. *et al.* Ca2+ and Mg2+ binding site engineering increases the degradation of polyethylene terephthalate films by polyester hydrolases from Thermobifida fusca. *Biotechnology Journal* **10**, 592-598 (2015).
2. Herrero Acero, E. *et al.* Enzymatic surface hydrolysis of PET: effect of structural diversity on kinetic properties of cutinases from Thermobifida. *Macromolecules* **44**, 4632-4640 (2011).
3. Sulaiman, S. *et al.* Isolation of a Novel Cutinase Homolog with Polyethylene Terephthalate-Degrading Activity from Leaf-Branch Compost by Using a Metagenomic Approach. *Appl. Environ. Microbiol.* **78**, 1556 (2012).
4. Xi, X. *et al.* Secretory Expression in Bacillus Subtilis and Biochemical Characterization of a Highly Thermostable Polyethylene Terephthalate Hydrolase from Bacterium HR29. *Enzyme Microb. Technol.* **143**, 109715 (2021).
5. Bollinger, A. *et al.* A novel polyester hydrolase from the marine bacterium Pseudomonas aestusnigri–structural and functional insights. *Frontiers in microbiology* **11**, 114 (2020).
6. Danso, D. *et al.* New Insights into the Function and Global Distribution of Polyethylene Terephthalate (PET)-Degrading Bacteria and Enzymes in Marine and Terrestrial Metagenomes. *Appl. Environ. Microbiol.* **84**, e02773-02717 (2018).
7. Sagong, H.-Y. *et al.* Structural and functional characterization of an auxiliary domain-containing PET hydrolase from Burkholderiales bacterium. *J Hazard Mater* **429**, 128267 (2022).
8. Sagong, H. Y. *et al.* Implications for the PET decomposition mechanism through similarity and dissimilarity between PETases from Rhizobacter gummiphilus and Ideonella sakaiensis. *J Hazard Mater* **416**, doi:ARTN 12607510.1016/j.jhazmat.2021.126075 (2021).
9. Yoshida, S. *et al.* A Bacterium That Degrades and Assimilates Poly(Ethylene Terephthalate). *Science* **351**, 1196 (2016).
10. Wei, R. *et al.* Functional characterization and structural modeling of synthetic polyester-degrading hydrolases from Thermomonospora curvata. *AMB express* **4**, 1-10 (2014).
11. Müller, R. J., Schrader, H., Profe, J., Dresler, K. & Deckwer, W. D. Enzymatic Degradation of Poly(Ethylene Terephthalate): Rapid Hydrolyse Using a Hydrolase from T. Fusca. *Macromol. Rapid Commun.* **26**, 1400 (2005).
